# Over-expressed lncRNA HOTAIRM1 promotes tumor growth and invasion through up-regulating HOXA1 and sequestering G9a/EZH2/Dnmts away from the *HOXA1* gene in glioblastoma multiforme

**DOI:** 10.1186/s13046-018-0941-x

**Published:** 2018-10-30

**Authors:** Qi Li, Chengya Dong, Jiayue Cui, Yubo Wang, Xinyu Hong

**Affiliations:** 1 China National Clinical Research Center for Neurological Diseases, Beijing Tian Tan Hospital, Capital Medical University, 6 Tiantan Xili, Dongcheng District, Beijing, 100050 China; 2 Department of Histology and Embryology of Basic Medicine College, Jilin University, Changchun, Jilin Province China; 3 Department of Neurosurgery, The First Hospital of Jilin University, 71 Xinmin Street, Changchun, 130021 Jilin Province China

**Keywords:** Glioblastoma multiforme, LncRNA, HOTAIRM1, *HOXA1* gene, Epigenetic regulation

## Abstract

**Background:**

Glioblastoma multiforme (GBM) is the common primary brain tumor classified the most malignant glioma. Long non-coding RNAs (LncRNAs) are important epigenetic regulators with critical roles in cancer initiation and progression. LncRNA HOTAIRM1 transcribes from the antisense strand of *HOXA* gene cluster which locus in chromosome 7p15.2. Recent studies have shown that HOTAIRM1 is involved in acute myeloid leukemia and colorectal cancer. Here we sought to investigate the role of HOTAIRM1 in GBM and explore its mechanisms of action.

**Methods:**

The expressions of HOTAIRM1 and HOXA1 in GBM tissues and cells were determined by qRT-PCR, and the association between HOTAIRM1, HOXA1 transcription and tumor grade were analyzed. The biological function of HOTAIRM1 in GBM was evaluated both in vitro and in vivo. Chromatin immunoprecipitation (ChIP) assay and quantitative Sequenom MassARRAY methylation analysis were performed to explore whether HOTAIRM1 could regulate histone and DNA modification status of the *HOXA1* gene transcription start sites (TSS) and activate its transcription. ChIP and RNA-ChIP were further performed to determine the molecular mechanism of HOTAIRM1 in epigenetic regulation of the *HOXA1* gene.

**Results:**

HOTAIRM1 was abnormally up-regulated in GBM tissues and cells, and this up-regulation was correlated with grade malignancy in glioma patients. HOTAIRM1 silencing caused tumor suppressive effects via inhibiting cell proliferation, migration and invasion, and inducing cell apoptosis. In vivo experiments showed knockdown of HOTAIRM1 lessened the tumor growth. Additionally, HOTAIRM1 action as regulating the expression of the *HOXA1* gene. HOXA1, as an oncogene, it’s expression levels were markedly elevated in GBM tissues and cell lines. Mechanistically, HOTAIRM1 mediated demethylation of histone H3K9 and H3K27 and reduced DNA methylation levels by sequester epigenetic modifiers G9a and EZH2, which are H3K9me^2^ and H3K27me^3^ specific histone methyltransferases, and DNA methyltransferases (DnmTs) away from the TSS of *HOXA1* gene.

**Conclusions:**

We investigated the potential role of HOTAIRM1 to promote GBM cell proliferation, migration, invasion and inhibit cell apoptosis by epigenetic regulation of *HOXA1* gene that can be targeted simultaneously to effectively treat GBM, thus putting forward a promising strategy for GBM treatment. Meanwhile, this finding provides an example of transcriptional control over the chromatin state of gene and may help explain the role of lncRNAs within the *HOXA* gene cluster.

**Electronic supplementary material:**

The online version of this article (10.1186/s13046-018-0941-x) contains supplementary material, which is available to authorized users.

## Background

Glioblastoma multiforme (GBM) is the most common and primary malignant tumor in the central nervous system with high invasive and excessive proliferative feature, and easy to recurrence. According to the pathological histology, the World Health Organization (WHO) divided primary brain tumors into four levels: grade I-IV and GBM is the highest severity glioma (grade IV) [1]. Prognosis for GBM patients is poor with overall survival of only 12–15 months for those patients who had the maximal safe resection and following radiotherapy and chemotherapy, and even lower for those where surgery is contraindicated [2, 3]. In recent years, molecularly targeted therapy has been a research hotspot in GBM treatment with its specificity and efficacy, however, the molecular heterogeneity and pathogenesis of GBM are not well understood [4]. Therefore, understanding the molecular mechanisms associated with the GBM development is critical, where long non-coding RNAs (LncRNAs) are promising candidates.

Protein-coding genes only account for 1–2% of the human genome, whereas the vast majority of transcripts are non-coding RNAs, and lncRNAs are a class of RNAs with transcripts longer than 200 nucleotides and have little or no protein-coding potential [5]. Deregulation of lncRNAs impacts different cellular processes of the tumor, such as cell proliferation, migration, invasion, and apoptosis; therefore, lncRNAs may serve as either oncogenes or cancer suppressor genes in tumorigenesis and tumor progression [6, 7]. LncRNAs are key regulators of chromatin structure, affecting epigenetic states and expression levels of various target genes through interactions with histone modifiers, chromatin remodeling complexes, transcriptional regulators, or the DNA methylation machinery [8, 9]. Recent reports demonstrate that lncRNAs play an important role of epigenetic gene regulation in GBM, for example, NEAT1 caused of *ICAT*, *GSK3B* and *Axin2* genes silencing through interacting with EZH2 and mediating H3K27me^3^ increase, and promoted GBM cell growth and invasion, and then contributed to GBM progression [10]. HOTAIR regulated cell cycle through interacting with EZH2 and affected tumor growth of GBM in vivo and in vitro [11].

LncRNA, HOXA transcript antisense RNA myeloid-specific 1 (HOTAIRM1), locates in the 5′ end of *homeobox A* (*HOXA*) gene cluster, is a natural antisense transcript of *HOXA1* gene and expresses in the myeloid lineage [12] and induced during neuronal differentiation [13]. HOTAIRM1 plays a key role during myeloid maturation and highly expresses in acute myeloid leukemia, which impacts the prognosis of patients [14, 15]. Recently, HOTAIRM1 was found lowly expressed in tissues and plasma of colorectal cancer and may be as a potential biomarker for diagnosis of colorectal cancer [16]. The HOTAIRM1 expression is more ubiquitous, and HOTAIRM1 was found to be a low expression in the adult brain, but highly expressed in the fetal brain [13]. A fetal lncRNA that is reactivated in cancer malignant progression may represent a critical regulator of cellular growth, differentiation. But the exact roles of HOTAIRM1 in GBM remain unclear.

In the present study, we detected the expression pattern, functional role and underlying mechanisms of HOTAIRM1 in GBM. After HOTAIRM1 silencing cell proliferation, apoptosis, migration, invasion and tumor growth in vivo were assessed, which implied HOTAIRM1 might exert oncogenic properties in GBM. More importantly, HOTAIRM1 could interact with EZH2, G9a and DNA methyltransferases (Dnmts) and sequester them away from the transcription start sites (TSS) of *HOXA1* gene, thereby activating the *HOXA1* oncogene expression. This finding reveals a novel mechanism by with HOTAIRM1 mediated GBM proliferation and invasion and demonstrates that HOTAIRM1 may be a promising target for the GBM treatment.

## Methods

### Cell culture

Established human GBM cell lines (U87, U251, and A172) were grown in Dulbecco’s modified Eagle’s medium (DMEM, GIBICO, Carlsbad, CA, USA) supplemented with 10% fetal bovine serum (FBS, HyClone, Logan, UT, USA). Primary patient-derived GBM cells (G0410, G0515, and G0923) were cultured in Minimum Essential Medium (MEM Alpha) (GIBICO) supplemented with 20% FBS. Human Astrocytes (HA) cell were grown in Astrocytes Medium (ScienCell, San Diego, California, USA) with 2% FBS, 1% astrocyte growth supplement and 1% penicillin/streptomycin.

### Patients and samples

Seventy glioma and 20 normal brain samples were obtained from the Department of Neurosurgery, The First Hospital of Jilin University (Changchun, Jilin, China). All tissues samples were frozen in liquid nitrogen immediately after resection and stored at liquid nitrogen until use. All clinical pathologic and biological data were available for those patients. The study was subjected to approval by the Ethical Committee of The First Hospital of Jilin University and informed consent was issued by all patients. All of the tumor tissues were obtained at primary resection, and none of the patients had undergone chemotherapy or radiation therapy prior to surgery.

### Primary derived GBM cells

Tumors were minced in PBS and digested in Hanks balanced salt solution (HBSS) containing 0.1% EDTA and 0.25% trpsin (GIBICO) for 30 min at 37 °C, and then cells were incubated with red cell lysis buffer (Sigma-Aldrich) to remove red blood cells. Cells were serially passed through 0.45 μm filters and plated in MEM Alpha supplemented with 20% FBS. Primary GBM samples (designated G0410, G0515, and G0923) were obtained from patients undergoing resection in accordance with a protocol approved by the Jilin University Medical Center Institutional Review Board.

### RNA preparation and quantitative real-time RT-PCR

Total RNA was extracted from cells and tissues using Trizol reagent (Invitrogen, Waltham, MA, USA). RNA was reverse-transcribed with a PrimeScript™ RT reagent Kit (Takara Biotechnology, Dalian, China) for cDNA synthesis and genomic DNA removal. Quantitative real-time PCR was carried out in the Takara real-time PCR system using SYBR® Premix Ex Taq™ II Kit (Takara). Housekeeping gene GAPDH was used as the endogenous control. The relative expression was calculated using the 2^-ΔΔ^Ct method. The expression of GADPH is stable in brain and glioma tissues and glioma cells, and GAPDH is suitable reference genes for expression analysis in human glioma using RT-qPCR [17]. The primers are listed in Additional file 1: Table S1. The primer amplification efficiency of HOTAIRM1 and HOXA1 was detected using cDNA template gradient dilution method and the amplification efficiency were 101.0% and 97.7% respectively, approaching 100% (Additional file 2: Figure S1A, B).

### RNA-fish

HOTAIRM1 probe were synthesized by BIOSEARCH Technology (Novato, CA, USA). The slides of A172 and U87 cells were fixed in 4% paraformaldehyde for 20 min, and digested with protein K at 37 °C for 10 min. Then the slides were washed with PBS twice and dehydrated by ascending series of ethanol. After denatured at 73 °C for 3 min, 20 μl hybridization reaction solution (2 μl probes+ 18 μl hybridization reaction) were added to the slides. The slides were hybridized at 42 °C overnight. After that, the slides were washed with 25% formamide/2 × Saline Sodium Citrate (SSC) at 53 °C twice and descending series of SSC at 42 °C. Finally, the slides were stained with DAPI and subjected to fluorescent signal detection using Zeiss LSM710 confocal laser microscopy (Zeiss, Germany). GBM tissues were fixed in 4% paraformaldehyde immediately after the operation. After 72 h, the tissues were dehydrated in graded sucrose, embedded in OCT compound (Sakura, Torrance, CA, USA), and frozen in − 80 °C. The frozen tissues were sliced at a thickness of 4–10 μm and mounted no a microscope slide, the slide-mounted tissue sections were subjected to FISH as the cell slides. All probe sequences of HOTAIRM1 were listed in Additional file 3: Table S2.

### Synthetic HOTAIRM1 siRNA knockdown

Transient knockdown of HOTAIRM1 in GBM cells was performed by siRNA transfection. siRNA oligonucleotides targeting HOTAIRM1 were designed and synthesized by Genepharma (Shanghai, China). The nonspecific siRNA oligonucleotides (Genepharma) were used as negative controls. All siRNA oligonucleotide sequences are listed in Additional file 4: Table S3. Cells were transfected with 150 pmol siRNA/well in 6-well plates, using Lipofectamine RNAiMax Reagent (Invitrogen) based on the manufacturer’s instructions.

### Cell counting kit-8 (CCK-8) assay, BrdU cell proliferation, and cell apoptosis analysis

The CCK-8 detection kit (Dojindo, Shanghai, China) was used to measure cell viability. Cells were seeded in a 96-well (100 μl per well) plates at a density of 5 × 10^4^/ml. CCK-8 solution (10 μl per well) was added and the plate was incubated at 37 °C for 2 h. The viable cells were counted by absorbance measurements with a monochromator microplate reader at a wavelength of 450 nm.

BrdU incorporation and cell proliferation analysis were detected using BD Pharmingen™ BrdU Flow Kit (BD, San Diego, CA, USA). Cells were plated into 6-well plates and transfected with siRNA for 24 h, added BrdU (1 mM/mL) for 12 h and then harvested. Cells were stained with anti-BrdU and 7-AAD according to the manufacturer’s instructions. Cell cycle distribution was determined by flow cytometry (FACSAriaTM II, Becton Dickinson, Mountain View, CA, USA).

The Annexin V-PE assay kit (BD) was utilized to analyze cell apoptosis in GBM cells. After transfected with siRNA for 24 h, cells were harvested and resuspended in 100 ul binding buffer at a density of 1 × 10^6^ cells/ml. PE-conjugated Annexin V and 7-AAD reagent staining were performed in concentrations and time recommended by the manufacturer. Stained cells were analyzed by flow cytometry (FACSAria II, BD).

### Lentiviral production and stable cell line establishment

A pMagic 7.1-shRNA-GFP lentiviral vector (Sbo-bio, Shanghai, China) was used for the shHOTAIRM1 knockdown experiment. Briefly, lentiviral particles expressing HOTAIRM1-specific shRNAs or pMagic7.1 vector were co-transfected into 293 T cells with the mixed set of packaging plasmids (SPAX2 and MD2G) using Lipofectamine 3000 (Invitrogen, Carlsbad, CA, USA). Lentiviral particles in 293 T cells were produced and the viruses were concentrated and titered. A172 and U87 cells were infected with the HOTAIRM1 shRNA construct, and GFP^+^ cells were sorted by flow cytometry. Then, cells were cultured with the regular complete medium. Finally, cells were tested for mRNA expression by qRT-PCR.

The shRNA sequences were used for HOTAIRM1 knockdown: shHOTAIRM1–1,CCGGGCCTCTATTACCAATTTAAATTCTCGAGTTAAATTGGTAATAGAGGCAGTTTTTTG; shHOTAIRM1–2, AATTCAAAAAACTGCCTC TATTACCAATTTAACTCGAGATTTAAATTGGTAATAGAGGC.

### Tumor xenografts model

Twenty five-week-old male nude mice were housed in specific pathogen-free environments. Mice were acclimated to the environment for 7 days before the experiments and they were randomly divided into two groups. Cultured U87 cells with pMagic 7.1 vector and U87 cells with pMagic 7.1-shRNA (3 × 10^6^ cells) were respectively injected subcutaneously into the right flanks of mice. Tumor volumes were calculated as V (mm^3^) = 0.5 × L × W × H. The tumor volumes of xenografts were monitored weekly.

### Cell scratch assay and transwell cell invasion assay

Cell migration was assessed with the scratch (wound healing) assay. Cells were seeded in 6-well plate to create a confluent monolayer. Then, the monolayer was scraped to make a wound using a p200 micro-pipette tip. The cells were washed twice with PBS and incubated in 0.1% FBS culture medium. Cells at multiple points along the scratch were imaged every 12 h period using a microscope.

Cell invasion was detected using transwell assay (8 μm pore size, Millipore, Darmstadt, Germany). 5 × 10^4^ cells in serum-free medium were planted into the upper chamber of an insert with matrigel. DMEM medium containing 20% FBS was added to the lower chamber. After incubation at 37 °C for 24 h, cells remaining on the upper membrane were removed with cotton wool. Cells that had migrated through the membrane were stained with 0.1% crystal violet, imaged.

### Chromatin immunoprecipitation (ChIP)

ChIP assay was performed with EZ-Magna ChIP A and EZ-Magna ChIP G Kits (Milliore) according to the manufacturer’s protocol. ChIP grade antibodies were as follows: anti- H3K27me^3^, anti- H3K9me^2^, anti-EZH2, normal rabbit IgG, and normal mouse IgG (Milliore); anti-G9a, anti-DnmT1, anti-DnmT3a, and anti-DnmT3b (Abcam). Immunoprecipitated DNA was analyzed by real-time PCR normalized with the input DNA. The sequences of the primers in reference to the TSS regions of the *HOXA1* gene are listed in Additional file 5: Table S4. The primer amplification efficiency of HOXA1 was detected using DNA template gradient dilution method and the amplification efficiency was 99.2%, approaching 100% (Additional file 2: Figure S1C).

### DNA extraction, DNA bisulfite modification, and quantitative Sequenom MassARRAY methylation analysis

Genomic DNA was extracted from the cells with a QIAamp DNA Mini Kit (Qiagen, Hilden, Germany), and the bisulfite conversion reaction was performed using an EpiTect Bisulfite kit (QIAGEN) according to the manufacturer’s instructions. The Sequenom MassARRAY platform (Oebiotech, Shanghai, China) was utilized to quantitatively analyze the methylation status of the *HOXA1* gene promoter. PCR primers (Additional file 6: Table S5) were designed using EpiDesigner. The PCR mixtures were pre-heated for 4 min at 94 °C, followed by 45 cycles of 94 °C for 20 s, 56 °C for 30 s and 72 °C for 60 s, the final extension at 72 °C for 3 min. PCR products were incubated with Shrimp Alkaline Phosphatase following the manufacturer’s protocol. After in vitro transcription and RNaseA digestion, small RNA fragments with CpG sites were acquired for the reverse reaction. The methylation ratios of the products were calculated using Epityper software Version 1.0 (Sequenom, San Diego, CA, USA).

### RNA-chromatin immunoprecipitations (RNA-ChIP) assays

RNA-ChIP assay was performed based on the described protocol [18, 19]. The RNA- ChIP assay was conducted without sonication using the following ChIP-grade antibodies: anti-EZH2 (Milliore); anti-G9a, anti-DnmT1, anti-DnmT3a, and anti-DnmT3b (Abcam). The immunoprecipitated RNA was purified using Trizol reagent and reverse transcription (Primer HOTAIRM1 A) was performed with the use of a PrimeScript™ RT reagent Kit (Takara), and the desired product was amplified by PCR (Primer listed in Additional file 7: Table S6).

### Statistical analysis

All data were expressed as the Mean ± standard deviation. All statistical analyses were done with Statistical Package for the Social Sciences (SPSS), version 17.0 (SPSS Inc., Chicago, IL, USA). The means between the two groups were compared using Student’s t-test and *p* < 0.05 was considered statistically significant.

## Results

### HOTAIRM1, a natural antisense lncRNA of *HOXA* gene cluster, up-regulates in GBM tissues and cells

**Fig. 1:**
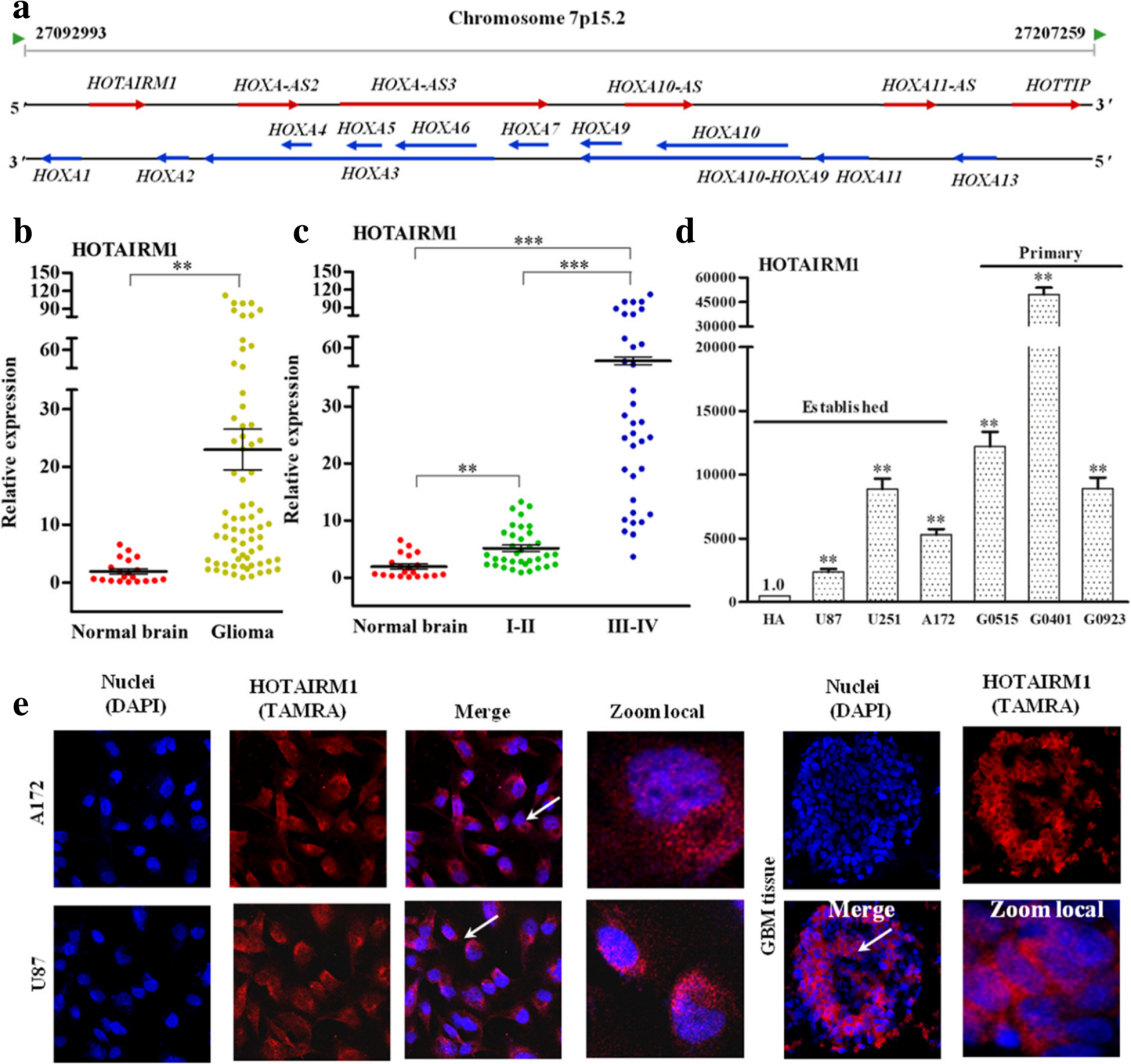
HOTAIRM1 is an antisense transcript to the *HOXA1* gene and up-regulates in glioma tissues and GBM cells. **a** Schematic representation of *HOXA* gene cluster on human chromosome 7p15.2. The RNA expression of HOTAIRM1 was analyzed using quantitative PT-PCR, **b** in normal brain and glioma (WHO I - IV) tissues; **c** in normal brain, low grade glioma (WHO I and II) and high grade glioma (WHO III and IV) tissues; **d** in HA cell, established and primary GBM cell lines; with the *GAPDH* gene as an internal control, and error bars represent the SEs of three independent experiments. **P < 0.01, *** *P* < 0.001. **e** RNA-FISH analysis of HOTAIM1 RNA in A172 and U87 cells, and GBM tissues, and photomicrograph with confocal laser microscopy (20×)

The human *HOXA* gene cluster (at 7p15.2) comprises 11 genes: *HOXA1–7*, *HOXA9–11*, and *HOXA13*. A GeneBank search identified 6 lncRNAs on the opposite strand of the *HOXA* gene cluster (Fig. 1a). The lncRNA--HOTAIRM1 is transcribed from the antisense strand of the *HOXA1* gene in a head-to-head orientation. First, we examined the expression levels of HOTAIRM1 in tumor tissues from 40 patients with glioma (WHO I and II, *n* = 20; WHO III and IV, *n* = 20) and 20 normal brain tissues. Our results showed that HOTAIRM1 was significantly up-regulated in glioma tissues compared with that in normal brain tissues (Fig. 1b). In addition, HOTAIRM1 expression was associated with the grade malignancy of brain tumor, and HOTAIRM1 levels of high-grade glioma (WHO III and IV) were significantly higher than low-grade glioma (WHO I and II) (Fig. 1c). We analyzed the association between HOTAIRM1 expression level with patient age, gender, tumor WHO clinical grade and found HOTAIRM1 expression level was related to tumor grade and was not related to age and gender (Table 1). Next, we screened a cohort of established GBM cell lines and primary patient-derived GBM cells for their HOTAIRM1 expression and showed that the majority of the GBM cell have elevated expression of HOTAIRM1 compared with human astrocytes (HA) (Fig. 1d). Finally, RNA FISH showed HOTAIRM1 localized both in the cytoplasm and nucleus of U87 and A172 cells, and this experimental result preliminarily suggests us that HOTAIRM1 is possible to regulate gene expression at transcriptional or post-transcriptional levels. And it also can be detected in GBM tissues (Fig. 1e).

**Table 1:** Demographic and clinical features of the patients with glioma

Characteristic	Glioma = 70	*P*
Gender		
Female	32	
Male	38	0.7686
Age		
≤ 60	34	
> 60	36	0.7643
WHO clinical grade		
I-II	35	
III-IV	35	0.0133

Thus, our results indicate that HOTAIRM1 highly expressed in the GBM tissues and cells and that it may be related to the GBM genesis.

### Knockdown of HOTAIRM1 inhibits GBM growth in vitro and in vivo

**Fig. 2:**
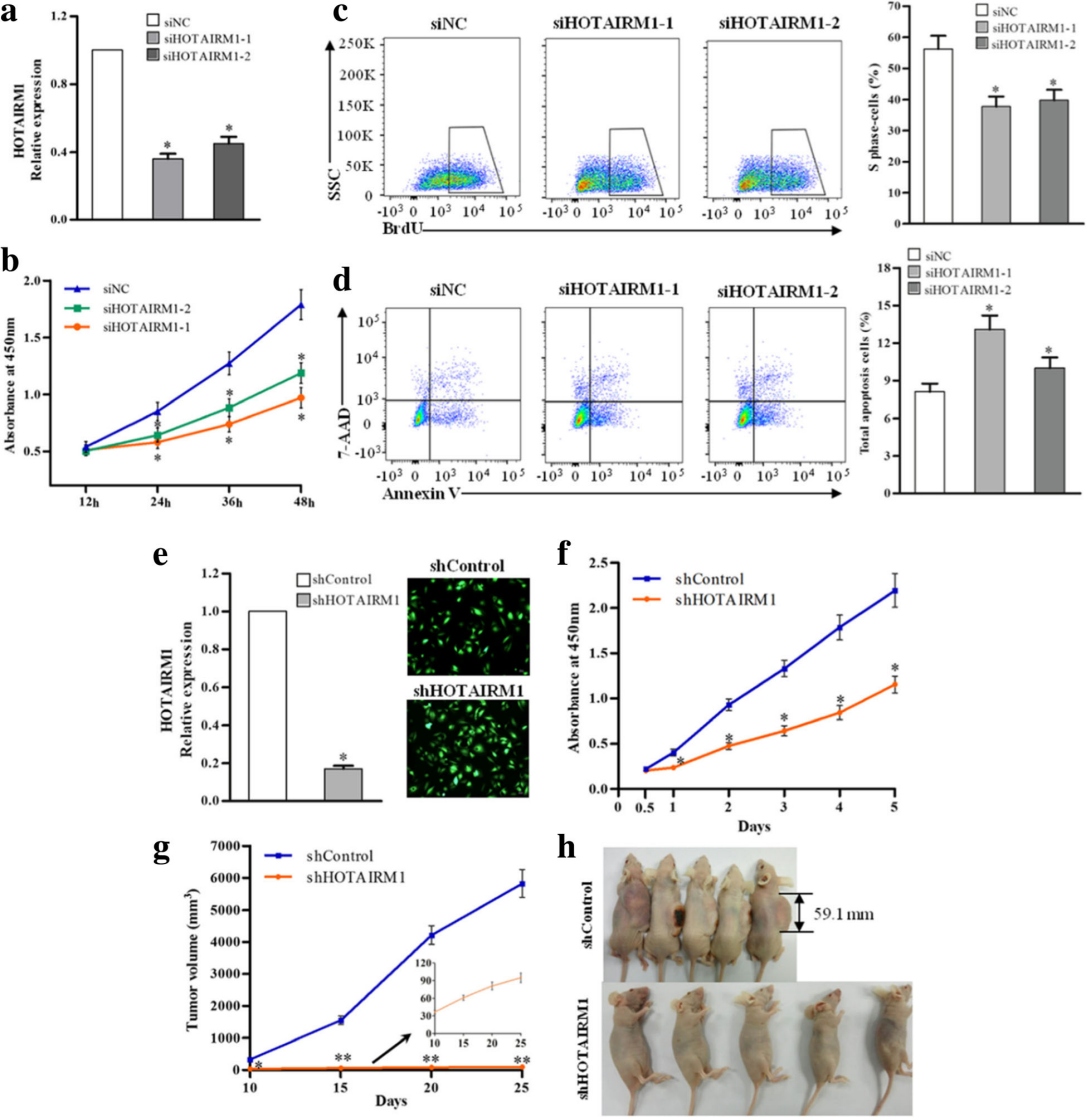
Knockdown of HOTAIRM1 suppresses proliferation and induces apotosis of GBM cells and inhibits GBM xenograft tumorigenesis in vivo. **a** The qRT-PCR analysis of HOTAIRM1 RNA levels at 24 h after siHOTAIRM1 treatment, with the *GAPDH* gene as an internal control. The siControl was a scrambled sequence with no homology to any known gene. After treatment A172 cell with siHOTAIRM1 and siNC for 24 h, **b** cell growth curve was determined by CCK-8 assay at various time points (12 to 48 h); **c** representative flow cytometry cell cycle profiles and the plot showing changes in cell proliferation; **d** flow cytometry analysis showing cells apoptosis rate. **e** The qRT-PCR analysis of HOTAIRM1 RNA levels after transfection with lentivirus of shHOTAIRM1 or shControl with the *GAPDH* gene as an internal control, and representative images shower cells with GFP^+^. **f** After transfection with shHOTAIRM1 or shControl, A172 cell growth curve was determined by CCK-8 assay at a different time point (0.5 to 5 day). **a**-**f** Error bars represent the SEs of three independent experiments, *P < 0.05. **g** The anti-tumor effect of knockdown of HOTAIRM1 in vivo. shHOTAIRM1/U87 cells and shControl/U87 cells (1× 10^6^ cells per mouse) were subcutaneously injected into nude mice. The mean tumor volumes were assessed at the indicated days, *P < 0.05, ** *P* < 0.01. **h** After 25 days, the mice were sacrificed. Representative nude mice from the shControl and shHOTAIRM1 groups

To explore the role of HOTAIRM1 in GBM, the effects of reduced expression of HOTAIRM1 on cell proliferation and apoptosis were investigated in three GBM cell lines. First, the HOTAIRM1 expression was repressed by RNA interference. We measured HORAIRM1 levels in A172 and U87 cells after treated with siHOTAIRM1–1 or siHOTAIRM1–2. The results indicated that both siHOTAIRM1–1 and siHOTAIRM1–2 caused visible reductions in HORAIRM1 levels, with a greater inhibitory effect of siHOTAIRM1–1 compared with siHOTAIRM1–2 in two cell lines (Fig. 2a and Additional file 8: Figure S2A). The CCK8 and BrdU cell proliferation assays indicated that cell growth and proliferation were reduced by the knockdown of HOTAIRM1 in GBM cells (Fig. 2b and c and Additional file 8: Figure S2B-C). A significant increase of cell apoptosis was observed in HOTAIRM1-inhibiting GBM cell lines (Fig. 2d and Additional file 8: Figure S2D).

Next, the A172, U87, and G0401 cells were infected with the lentivirus containing shHOTAIRM1 vector showed a significant decrease of HOTAIRM1 expression levels compared with shControl vector (Fig. 2e, Additional file 8: Figure S2E and Additional file 9: Figure S3A). The growth curves determined by CCK8 assays indicated that knockdown of HOTAIRM1 dramatically suppressed the growth of GBM cells (Fig. 2f, Additional file 8: Figure S2F and Additional file 9: Figure S3B). A significant decrease in cell proliferation and the increase of cell apoptosis were observed in shHOTAIRM1-transfected G0401 cells (Additional file 9: Figure S3C and D).

At last, to investigate the effect of HOTAIRM1 on GBM growth in vivo, we subcutaneously injected U87 cells stably transfected with shHOTAIRM1 or shControl into nude mice for xenoplantation. Due to A172 cells don’t form tumors in vivo, in this study we choose the U87 cell line for xenograft experiment. As showed in Fig. 2g and h, the tumor growth of mice injected cells transfected with shHOTAIRM1 were significantly decreased compared with those injected cells transfected with shControl.

Taken together, these data support an important promoting role for HOTAIRM1 in GBM tumor growth in vitro and in vivo.

### HOTAIRM1 promotes migration and invasion of GBM cells

**Fig. 3:**
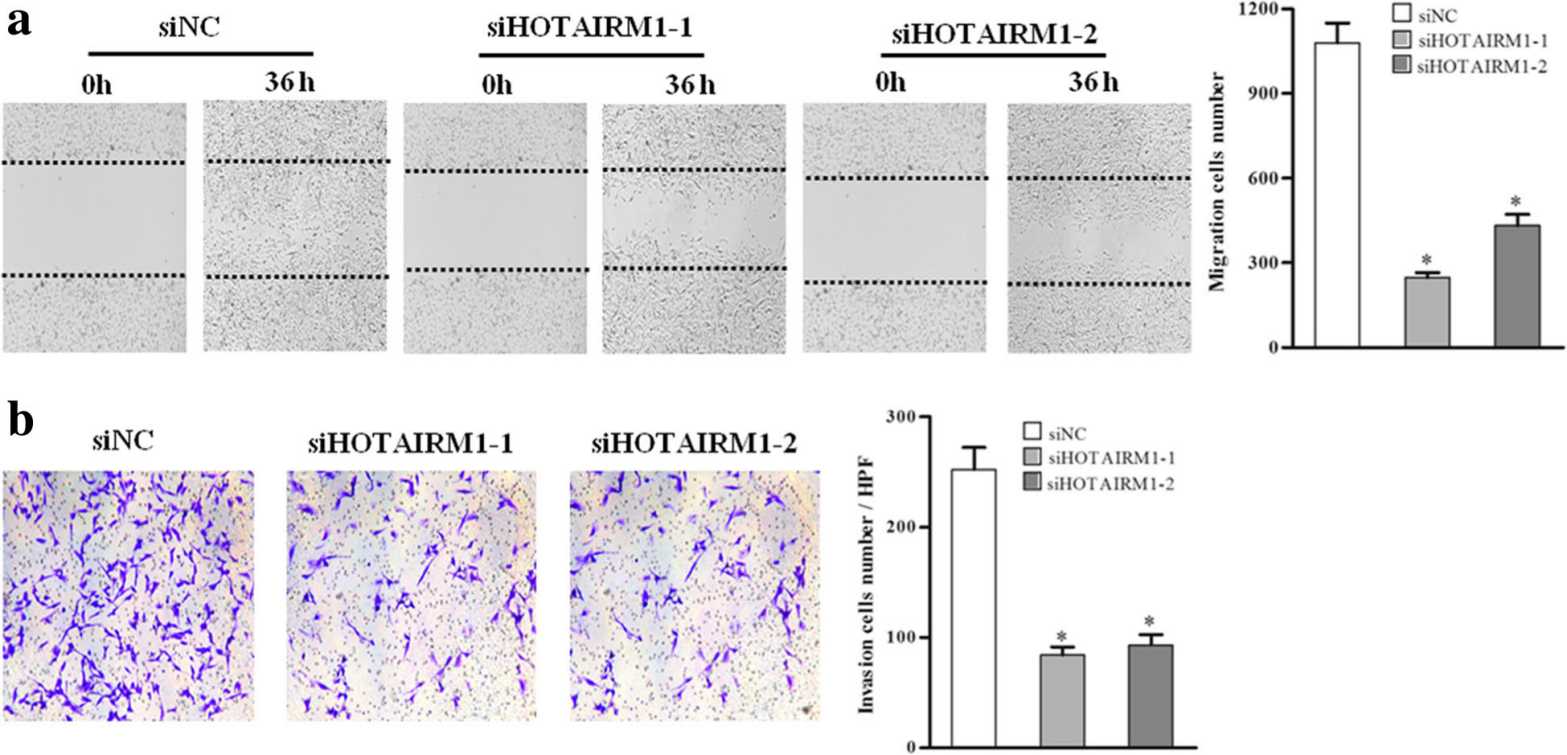
Knockdown of HOTAIRM1 inhibits migration and invasion of GBM cells. A172 cells were treated with siHOTAIRM1 or siNC for 24 h, **a** cell migration was observed under the microscope and migrated cell number was counted; **b** transwell cell invasion assay was performed, the invasive cells in members were stained with crystal violet and quantification under high-power field (HPF). Representative images were shown and a bar figure was presented. Error bars represent the SEs of three independent experiments, *P < 0.05

GBM shows highly aggressive biological character. To investigate whether HOTAIRM1 affected GBM cell migration and invasion, wound healing and transwell invasion arrays were performed. The results showed HOTAIRM1 knockdown significantly inhibited migration and invasion capacity of GBM cells, compared with control group (Fig. 3 and Additional file 10: Figure S4). These results indicate that HOTAIRM1 promote GBM cells migration and invasion.

### HOTAIRM1 regulates *HOXA1* gene expression

**Fig. 4:**
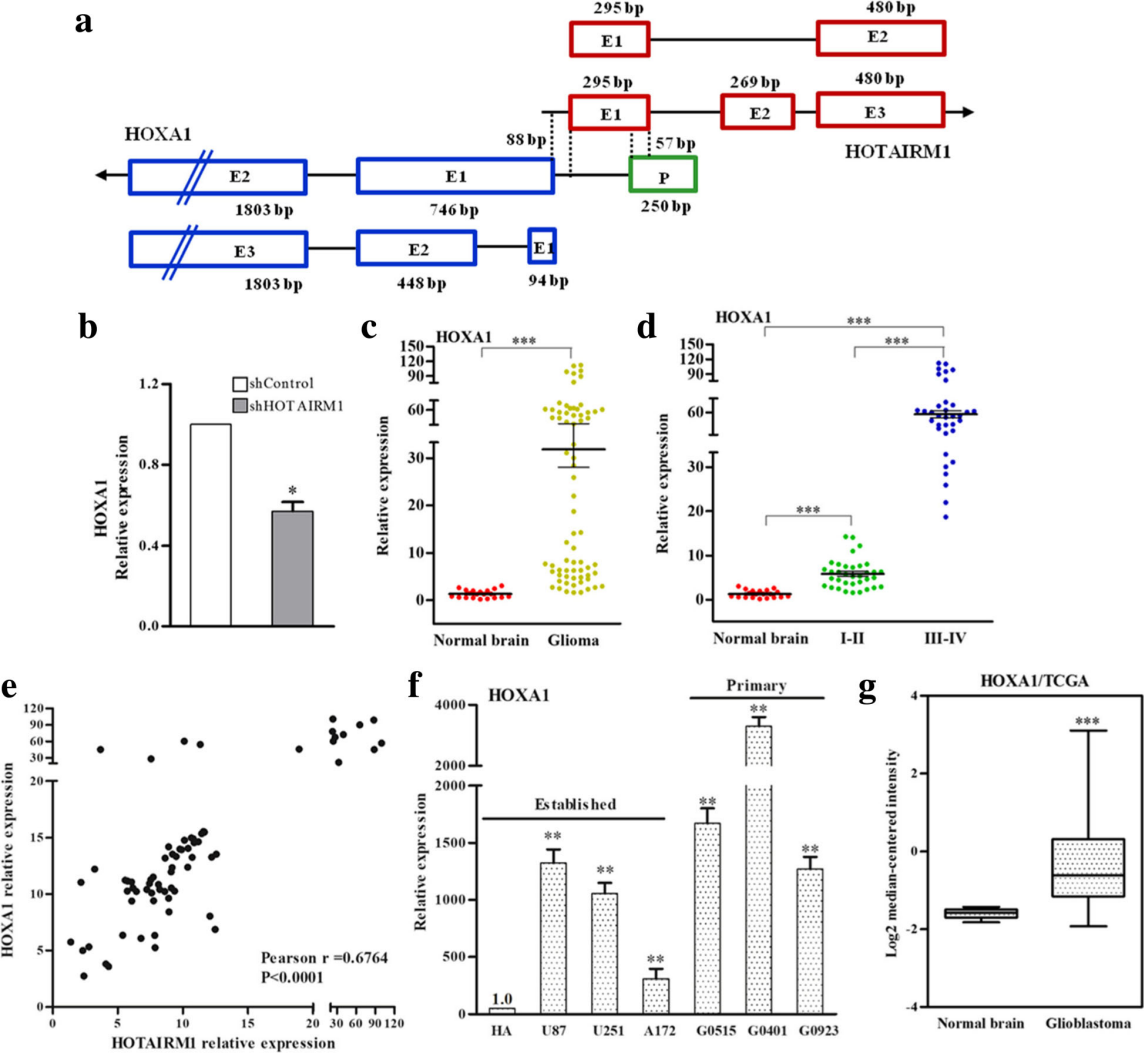
HOTAIRM1 regulates HOXA1 RNA levels in GBM cells. **a** The exon composition of HOTAIRM1 and HOXA1 transcript and organization of the overlapping region of them. The RNA expression of HOXA1 was analyzed using qRT-PCR, **b** after transfection with shHOTAIRM1 or shControl in A172 cells; **c** in normal brain and glioma (WHO I - IV) tissues; **d** in normal brain, low grade glioma (WHO I and II), and high grade glioma (WHO III and IV) tissues, with the *GAPDH* gene as an internal control, *** *P* < 0.001. **e** The correlational analyses between HOTAIRM1 and HOXA1 RNA expression levels in GBM tissues. **f** qPT-PCR analysis of the RNA levels of the *HOXA1* gene in HA cell, established and primary GBM cell lines. The qPCR was performed with the *GAPDH* gene as an internal control and error bars represent the SEs of three independent experiments, *P < 0.05, **P < 0.01. **g** Box plots depicting the mRNA levels of HOXA1 from 523 GBM patients and 10 normal brain tissues obtained from The Cancer Genome Atlas (TCGA), *** *P* < 0.001

LncRNAs have been implicated in the regulation of their neighboring sense gene expression. HOTAIRM1 have two alternative variants, variant 1 and variant 2 respectively consisting of three exons and two exons. Sequence analysis revealed that variant 1 and variant 2 commonly use the first and last exon, and variant 1 adds an exon between these two exons compared with variant 2. Exon 1 of HOTAIRM1 overlaps with the promoter of HOXA1 by 57 bp in an antisense fashion. Predicts promoter region was done using the PROSCAN Version 1.7 based on scoring homologies with putative eukaryotic Pol II promoter sequences (Fig. 4a). To check whether the HOTAIRM1 plays a regulatory role in gene expression at the *HOXA1* gene, we analyzed the changes in HOXA1 mRNAs levels, after silencing of HOTAIRM1. Introduction of shHOTAIRM1 resulted in decreasing in HOXA1 mRNA levels (Fig. 4b and Additional file 11: Figure S5A, B). We also investigate the changes in mRNA levels for other genes in *HOXA* gene cluster after silencing of HOTAIRM1 by qRT-PCR method. The result indicates knockdown of HOTAIRM1 had no effect on other HOXA genes expression at the transcriptional level (Additional file 11: Figure S5C). These results indicate that the antisense lncRNA of HORAIRM1 regulates *HOXA1* gene expression in *cis*.

To investigate the expression levels of HOXA1 in GBM, we examined the HOXA1 mRNA levels in tumor tissues from 40 patients with glioma (WHO I and II, *n* = 20; WHO III and IV, n = 20) and 20 normal brain tissues using qPCR. Our results showed that HOXA1 expression levels were significantly up-regulated in glioma tissues compared with that in normal brain tissues, and HOXA1 expression levels were associated with the grade malignancy of glioma (Fig. 4c and d). Statistical analysis showed strong and positive correlations between the *HOXA1* gene and HOTAIRM1 expression (Fig. 4e). To further confirm whether this change was common and consistent in GBM cells, we analyzed HOXA1 expression in three GBM cell lines, three primary GBM cells and HA cells. Our results showed that HOXA1 mRNA levels were up-regulated in GBM cells compared with that in HA cells (Fig. 4f).

Furthermore, we observed HOXA1 expression in the large cohorts of GBM patients available from The Cancer Genome Atlas (TCGA) database, data showed that HOXA1 were significantly increased in GBM (from 523 patients) compared with that in 10 normal brain tissues (Fig. 4g). Thus, we concluded that the increased expression of HOXA1 may play an important role in GBM progression and development.

### HOTAIRM1 reduces gene-suppressive histone H3K9me^2^ and H3K27me^3^ modifications in the TSS regions of the *HOXA1* gene

**Fig. 5:**
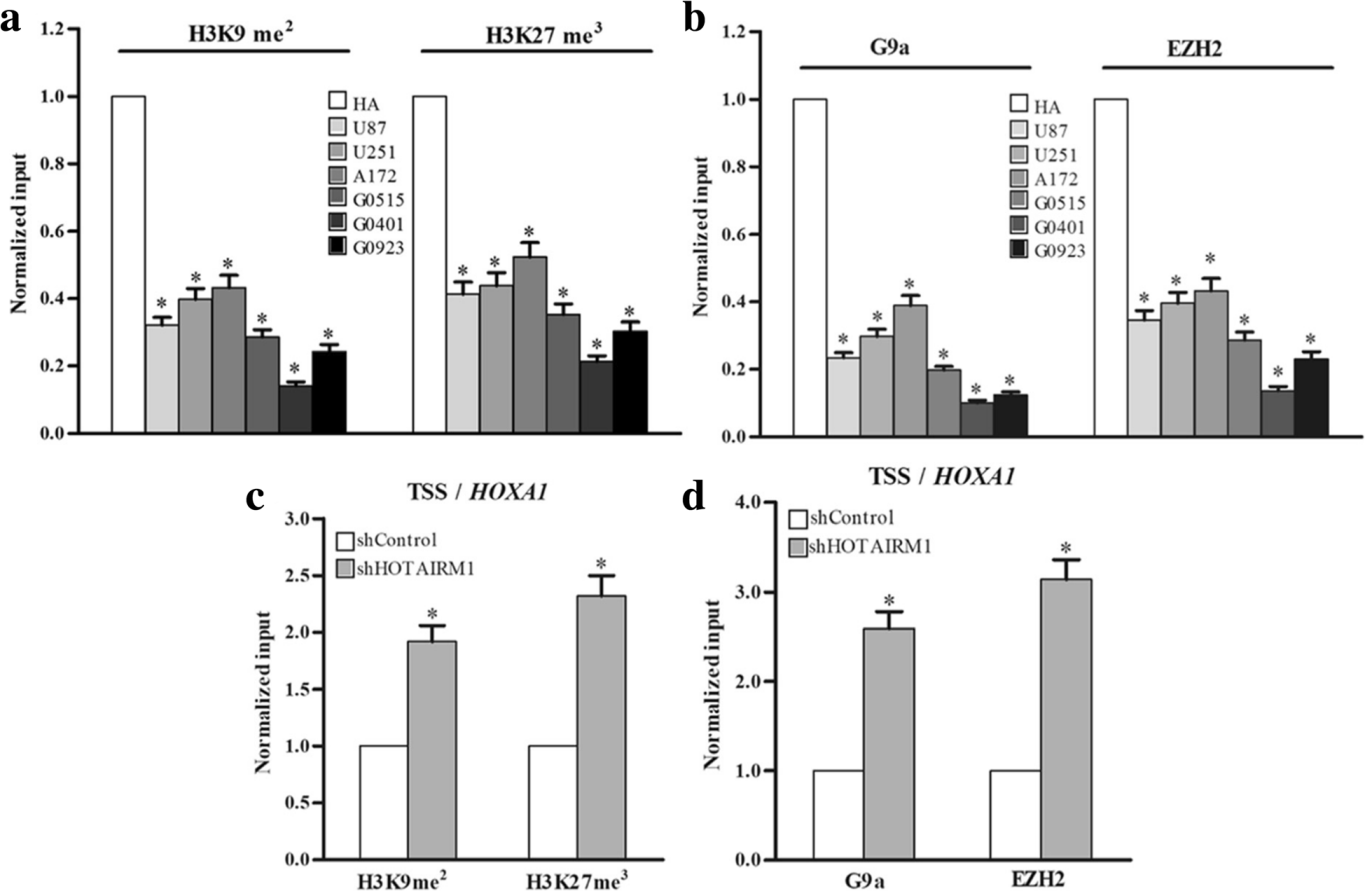
HOTAIRM1 decreases histone H3K9me^2^ and H3K27me^3^ modifications in the TSS region of the *HOXA1* gene in GBM cells. ChIP analysis of **a** histone H3K9me^2^ and H3K27me^3^ modifications; **b** G9a and EZH2 enrichment in the *HOXA1* gene TSS in A172 cells. Knockdown of HOTAIRM1, ChIP analysis of **c** histone H3K9me^2^ and H3K27me^3^ modifications; **d** G9a and EZH2 enrichment in the *HOXA1* gene TSS in A172 cells. ChIP enrichment was measured using qPCR, normalized by the input DNA, Error bars represent the SE of three independent experiments, *P < 0.05

To identify a possible mechanism of HOTAIRM1-mediated transcriptional promotion, we analyzed histone modification status in the TSS region of the *HOXA1* gene using a ChIP assay. In our first ChIP assays, using antibodies against H3K9me^2^, and H3K27me^3^, we found H3K9me^2^ and H3K27me^3^ modifies in the HOXA1 TSS in GBM cell lines and primary GBM cells were significantly decreased compared with that in HA cells (Fig. 5a). We next confirmed the enrichment of histone H3K9me^2^ methyltransferase G9a and H3K27me^3^ methyltransferase EZH2 in the TSS regions of HOXA1 in HA cells were higher than that in GBM cells (Fig. 5b). EZH2 is an H3K27-specific histone methyltransferase that is a component of PRC2. G9a is responsible for monomethylation and dimethylation of H3K9, and H3K9me^2^ and H3K27me^3^ are important modifications for gene silencing.

We performed identical ChIP assays after knockdown of HOTAIRM1, H3K9me^2^ and H3k27me^3^ modifications were increased in the HOXA1 TSS regions in A172, U87, and G0401 cells (Fig. 5c and Additional file 12: Figure S6A and B). Moreover, our ChIP assay results confirmed that G9a and EZH2 were also enriched in the TSS region of the *HOXA1* gene in HOTAIRM1-inhibiting GBM cell (Fig. 5d and Additional file 12: Figure S6C and D).

At last, we examined H3K9me^2^/H3K27me^3^ and G9a/EZH2 enrichment using ChIP assay in the different regions of the *HOXA1* gene TSS + 2000 bp, and in the TSS region of *HOXA2*, *HOXA11* gene after knockdown of HOTAIRM1 (Additional file 12: Figure S6E). *HOXA2* is the closest gene to *HOXA1* and *HOXA11* is located downstream of the *HOXA* gene cluster. The H3K9me^2^/H3K27me^3^ and G9a/EZH2 enrichment increased in 3 regions (HOXA1–1, A1–2, A1–3) near to HOXA1 TSS, especially in predicted promoter region (HOXA1–2), whereas the change of enrichment was not detected in other 2 regions (HOXA1–4, 1–5) and in *HOXA2*, *HOXA11* gene TSS regions (Additional file 12: Figure S6 F-I). So, we showed the result of the HOXA1–2 fragment in *HOXA1* gene TSS regions in our ChIP experiment.

These results indicated that silencing of HOTAIRM1 reduced gene-suppressive histone modification H3K9me^2^ and H3K27me^3^ in the TSS region of the *HOXA1* gene, thereby decreasing HOXA1 mRNA expression level.

### HOTAIRM1 decreases DNA methylation levels in the promoter region of the *HOXA1* gene by reducing DNA methyltransferases

**Fig. 6:**
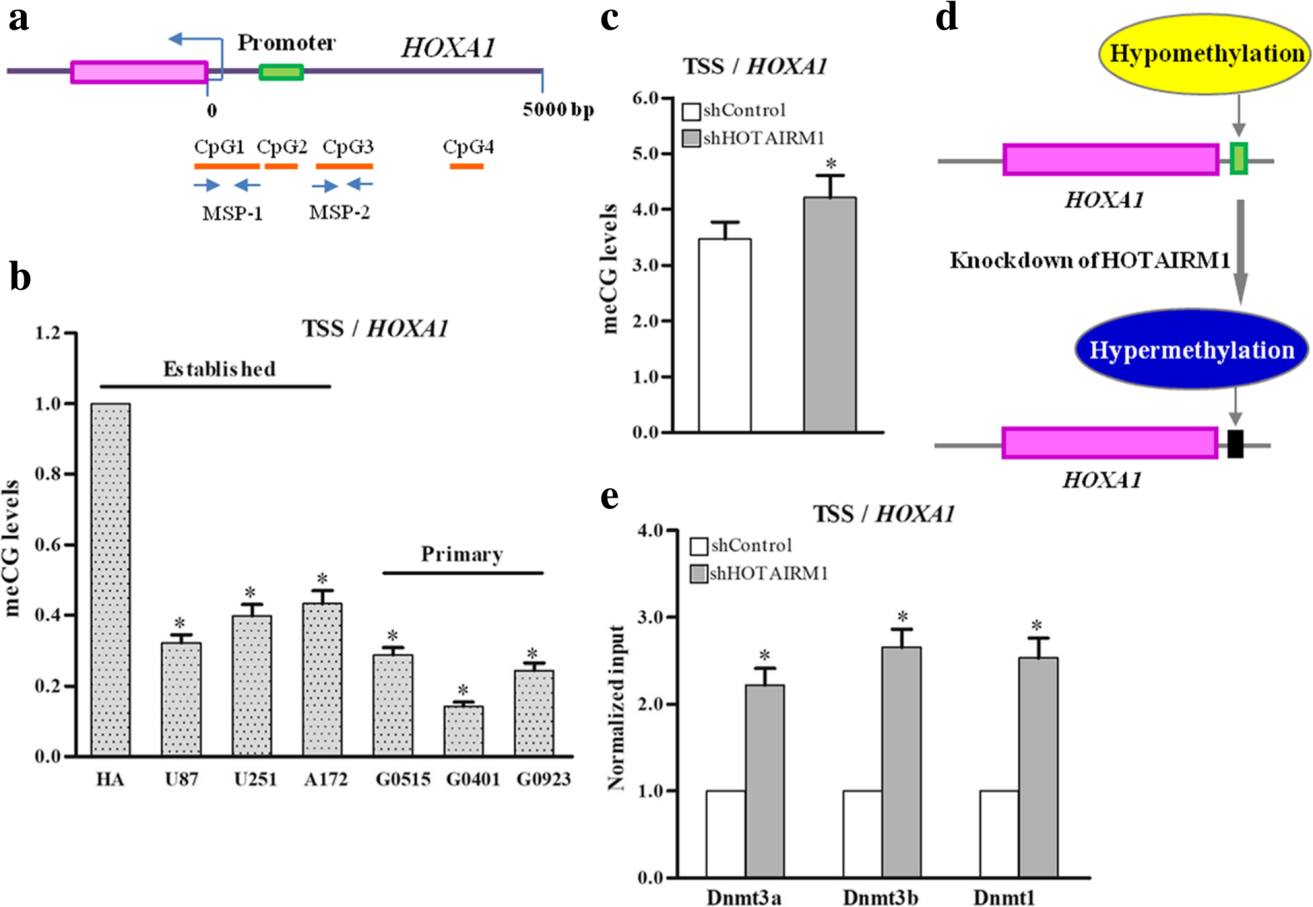
Knockdown of HOTAIRM1 induces CpG island methylation in the TSS region of the *HOXA1* gene by increasing DNA demethyltransferases. **a** The location of CpG island in the TSS region of the *HOXA1* gene and PCR segments of methylation specific PCR (MSP). The meCG levels in the *HOXA1* gene were measured to analyze DNA methylation status **b** in HA cell, established and primary GBM cell lines; **c** after knockdown of HOTAIRM1 in A172 cells. **d** The model for transitioning from a hypomethylated status to a hypermethylated status in the TSS region of *HOXA1*gene. The green and black box mark the predicted promoter regions of the *HOXA1* gene. **e** After knockdown of HOTAIRM1, ChIP analysis of DNA methyltransferase binding in the HOXA1 TSS region in A172 cells. Error bars represent the SE of three independent experiments, *P < 0.05

Antisense lncRNA have been proposed to cause DNA methylation of sense gene. We examined the methylation status of two CpG islands in the *HOXA1* gene promoter of HA and GBM cells (Fig. 6a). The CpG sites of the *HOXA1* gene promoter were methylated in HA cell, correlating with the promoter’s transcriptionally repressed status, whereas the HOXA1 promoters were un-methylated in GBM cell lines and primary GBM cells, with *HOXA1* transcriptionally active (Fig. 6b). To analyze DNA methylation levels, we measured the ratio of methylated CpG to total CpG sites in promoter regions. After knockdown of HOTAIRM1, DNA methylation of *HOXA1* was significantly increased, changing the *HOXA1* promoter from a hypomethylated state to a hypermethylated state in GBM cell lines (Fig. 6c and d and Additional file 13: Figure S7A-C).

We then performed ChIP assays to detect the potential interaction between DnmTs and the *HOXA1* gene promoter after the HOTAIRM1-mediated knockdown. The results indicated that enrichments of DnmT1, DnmT3a, and DnmT3b in the promoter region of *HOXA1* gene were increased by shHOTAIRM1 in A172 (Fig. 6e) and U87, G0401 cells (Additional file 13: Figure S7D-E). To examine whether knockdown of HOTAIRM1 affected DNA methyltransferases binding another locus, we analyzed DnmTs enrichments in the *HOXA2* and *HOXA11* gene TSS region. We found the loss of HOTAIRM1 had no effect on DnmT1, DnmT3a, and DnmT3b bonding to the *HOXA2* and *HOXA11* gene, suggesting that HOTAIRM1 specifically regulates the HOXA1 DNA methylation levels (Additional file 13: Figure S7F).

Given that hypomethylated DNA is associated with active genes, whereas hypermethylated genes are silent, we conclude that the transcriptional activation of the *HOXA1* gene is regulated in part by HOTAIRM1-directed DNA demethylation.

### HOTAIRM1 interacts with G9a, EZH2 and DNA methyltransferase to sequester them away from the TSS region of *HOXA1* gene and wipes off epigenetic silencing of the *HOXA1* gene

Previous studies have suggested that lncRNAs can sequester regulators from their nuclear targets in *cis*. Our ChIP assay results confirmed that HOTAIRM1 mediated *HOXA1* gene activation through reducing repressive chromatin modifications of H3K9me^2^, H3K27me^3^ and DNA methylation in GBM cells. Moreover, G9a, EZH2, and Dnmts were also enriched in the TSS regions of the *HOXA1* gene in HA cells.

**Fig. 7:**
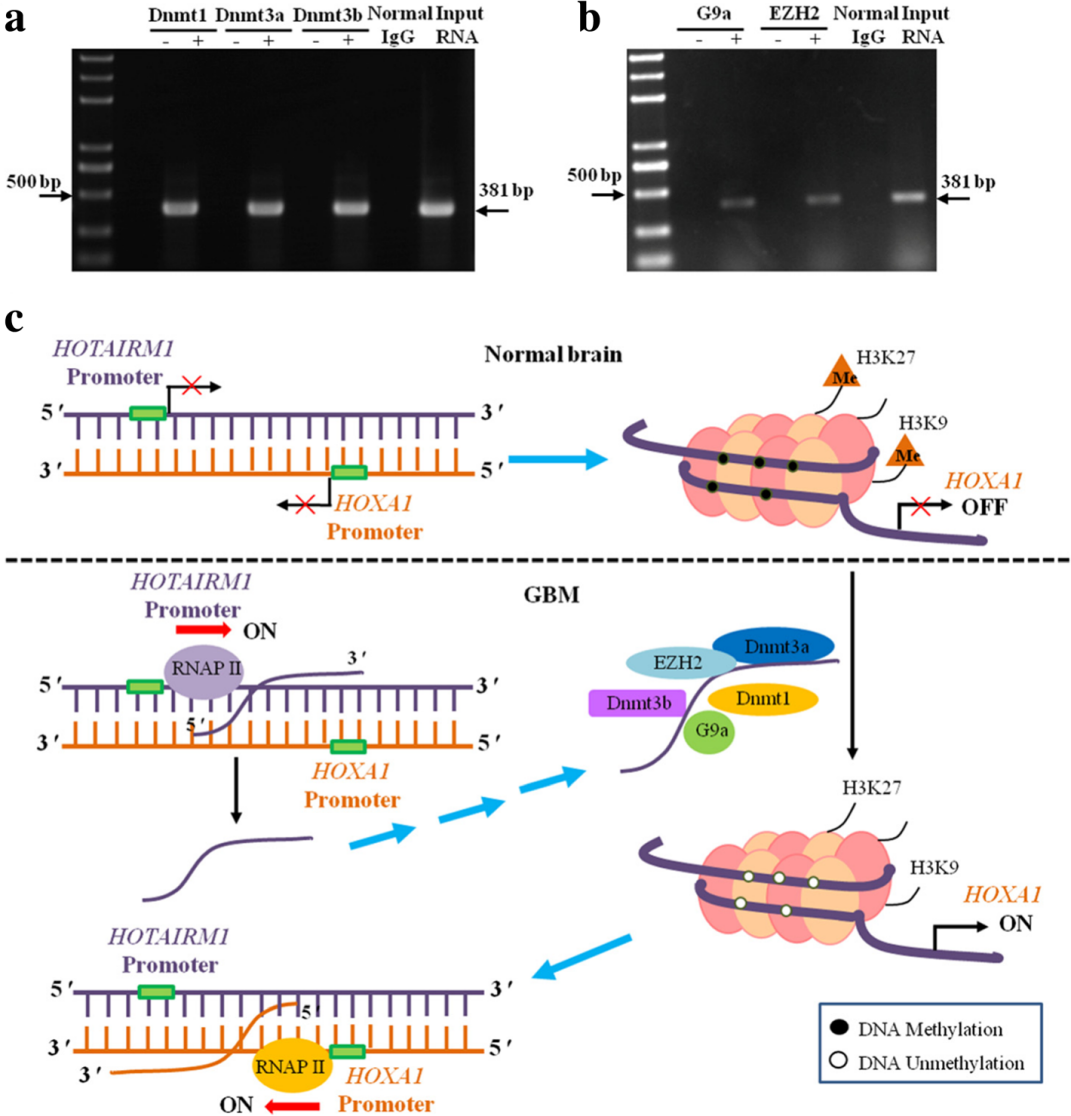
HOTAIRM1 interacts with repressive modifiers EZH2, G9a, and Dnmts to sequester them away from the promoter of *HOXA1* gene. **a** RNA-IP analysis of HOTAIRM1 interaction with histone methyltransferases EZH2, G9a and DNA methyltransferases Dnmt3a, Dnmt3b, Dnmt1 in A172 cells. One pair of primer was used to detect HOTAIRM1, the lane being 381 bp which represents the 3′ end of two variants. **b** A working model showing the possible role of HOTAIRM1 in the *HOXA1* gene regulation. In the normal brain, G9a, EZH2, and Dnmts enrichment lead to repressive histone modification H3K9me^2^, H3K27me^3^ and DNA methylation of the promoter of *HOXA1* gene, resulting in gene expression inhibition. In GBM cells, HOTAIRM1 interacts with G9a, EZH2, and Dnmts to sequester them away from the promoter of *HOXA1* gene. Histone H3K9, H3k27, and DNA are demethylated in the *HOXA1* promoter, changing the chromatin state into being open and active, and resulting in subsequent transcriptional activation

To analyze the epigenetic regulation mechanism of HOTAIRM1 mediated, we first tested whether HOTAIRM1 interacts with G9a, EZH2, and Dnmts by performing RNA-ChIP assays. In GBM cells, HOTAIRM1 was pulled down by either antibody (Fig. 7a and b), suggesting that HOTAIRM1 formed a complex with G9a, EZH2, and Dnmts and prevented them from binding the TSS of the *HOXA1* gene loci. This finding is consistent with the histone modifications of H3K9me^2^, H3K27me^3^ and DNA methylation levels in the *HOXA1* gene domains.

Based on our present results, we present a schematic model to illustrate the regulatory roles of HOTAIRM1 in the expression of the *HOXA1* gene (Fig. 7c).

## Discussion

In recent years, by RNA sequencing and annotation of the GENECODE project, thousands of lncRNAs have been discovered, but the functions of which have not been established. To discover novel tumor-related lncRNAs and determine their correlations with glioma subtypes, Zhang et al., used lncRNA classification pipeline to identify 1970 lncRNAs across different types and grades of human gliomas. Of these, HOTAIRM1 were up-regulated with increasing malignancy grades [20]. In this study, we validated high expressed HOTAIRM1 by quantitative RT-PCR in glioma tissues (grade I-IV) and GBM cell lines. HOTAIRM1 exhibited significantly increased levels in high-grade glioma compared to low-grade glioma and normal brains, indicating its potential roles in glioma biogenesis and development.

GBM is the most serious glioma (WHO IV). The aggressive nature and malignant proliferation of tumor cell are the major causes of death in patients with GBM. Therefore, identification of novel efficient method that can inhibit the growth and invasion of GBM is required. In this study, we demonstrated that knockdown of HOTAIRM1 inhibited GBM cell proliferation, migration, invasion and promoted cell apoptosis. Moreover, our in vivo experiments further showed tumor growth was effectively suppressed by HOTAIRM1 silencing. All these data indicated that HOTAIRM1 functioned as an oncogene in GBM. To our knowledge, this is first reported to investigate the function of HOTAIRM1 in GBM.

Identification of target genes is important for exploring the molecular mechanisms underlying HOTAIRM1 function. We verified HOTAIRM1 markedly regulated the expression of *HOXA1* gene. Currently, it has been demonstrated that HOTAIRM1 interact with Polycomb Repressive Complex 2 (PRC2) and histone demethylase UTX/MLL to regulate chromatin conformation and then affects *HOXA* gene cluster transcriptional activity [21]. Our research found that HOTAIRM1 activated transcription of the *HOXA1* gene through the decrease of histone H3K9me^2^, H3K27me^3^ and DNA methylation, which are epigenetic markers associated with gene silencing. While HOTAIRM1 interacting with G9a, EZH2 and DNA methyltransferases Dnmts, occlude them from the promoter of *HOXA1* gene, hence reducing their enrichment. This finding reveals an unexpected mechanism of gene control by lncRNA-mediated repressor occlusion.

The HOX family, a highly conserved family of genes encoding the class of transcription factors called homeobox genes, are found in clusters named HOXA, HOXB, HOXC, and HOXD, which are located on four separate chromosomes and consist of 9 to 11 genes arranged in tandem. Expression of these proteins is spatially and temporally regulated during embryonic development [22]. *HOXA* gene cluster encodes 11 DNA-binding transcription factors which may regulate gene expression, morphogenesis, and differentiation. There are 6 lncRNAs within the *HOXA* gene cluster, and several lncRNAs have been proposed to play key roles in glioma. Examples include HOXA11-AS, HOTTIP, HOXA-AS3, and HOXA-AS2. HOXA11-AS is transcribed from the 5-prime end of the HOXA transcript, which functions as miRNA sponge to promote the growth, migration, and invasion of glioma cells, and can serve as a biomarker of progression in glioma [23–25]. HOTTIP was aberrantly down-regulated in glioma, and over-expression of HOTTIP inhibited the growth of glioma in vitro and in vivo by regulation of BRE (brain and reproductive) gene expression, besides, HOTTIP promotes hypoxia-induced EMT of malignant glioma by regulating the miR-101/ZEB1 axis [26, 27]. Up-regulation of HOXA-AS3 promotes tumor progression and predicts poor prognosis in glioma [28]. HOXA-AS2 regulates malignant glioma behaviors and vasculogenic mimicry formation via the MiR-373/EGFR Axis [29]. Our study provides insight into how HOTAIRM1 regulated cell proliferation, migration, and invasion in GBM, and explain the precise transcriptional control of the *HOXA1* gene. Our results support the view that lncRNAs of *HOXA* gene cluster are the major player in GBM.

HOXA1, one of the *HOXA* gene cluster members, has been found to be up-regulated in human malignancies, such as non-small cell lung cancer [30], oral squamous cell carcinoma [31], uterine leiomyosarcoma [32] and breast cancer [33–35], and function as an oncogene. For example, the high expression level of HOXA1 promotes distant metastasis of melanoma [36]. In addition, elevated HOXA1 expression enhances cell proliferation, invasion, and metastasis in prostate cancer [37], and higher levels of HOXA1 correlates with accelerated cell proliferation and poor prognosis in gastric cancer [38]. Moreover, the *HOXA* gene cluster is aberrantly activated within confined chromosomal domains in GBM [39]. Our study showed that HOXA1 was up-regulated in GBM tissues and HOXA1 expression was positively correlated with HOTAIRM1 expression. As well as HOTAIRM1 controlled the local epigenetic status of *HOXA1* gene promoter and regulated *HOXA1* gene expression *in cis*. Thus, these findings suggest that the HOTAIRM1/HOXA1 axis may be involved in the initiation and development of GBM. However, elucidation of the downstream pathways involved in these processes should be investigated further.

## Conclusions

In summary, our current work revealed that the GBM-associated lncRNA HOTAIRM1 was an oncogenic factor that regulated *HOXA1* gene expression, promoting tumorigenesis by serving as a scaffold to sequester the chromosome modification enzyme G9a, EZH2 and DNA methyltransrase Dnmts away from the promoter of *HOXA1* gene. Thus, we highlighted that the HOTAIRM1/HOXA1 axis conferred an oncogenic function in GBM that might offer a novel therapeutic target.

## Additional files

Additional file 1: **Table S1.** Primers for qRT-PCR (DOCX 20 kb)

Additional file 2: **Figure S1.** QPCR primer line amplification efficiency detection. (DOCX 394 kb)

Additional file 3: **Table S2.** Probes Sequence for HOTAIRM1 (DOCX 20 kb)

Additional file 4: **Table S3.** siRNA oligonucleotides (DOCX 19 kb)

Additional file 5 **Table S4.** Primers for ChIP (DOCX 19 kb)

Additional file 6: **Table S5.** Primers for methylation specific PCR (DOCX 18 kb)

Additional file 7: **Table S6.** Primers for RNA-ChIP (DOCX 18 kb)

Additional file 8: **Figure S2.** Knockdown of HOTAIRM1 suppresses proliferation and induces apotosis of U87 cells. (DOCX 316 kb)

Additional file 9: **Figure S3.** Knockdown of HOTAIRM1 suppresses proliferation and induces apotosis of established and primary GBM cells. (DOCX 202 kb)

Additional file 10: **Figure S4.** Knockdown of HOTAIRM1 inhibits migration and invasion of established and primary GBM cells. (DOCX 203 kb)

Additional file 11: **Figure S5.** HOTAIRM1 regulates HOXA1 RNA levels in established and primary GBM cells. (DOCX 297 kb)

Additional file 12: **Figure S6.** Knockdown of HOTAIRM1 increased H3K9me2 and H3K27me3 modifications in the promoter region of the HOXA1 gene in established and primary GBM cells. (DOCX 758 kb)

Additional file 13: **Figure S7.** Knockdown of HOTAIRM1 induces CpG island methylation in the promoter region of the HOXA1 gene by increasing DNA demethyltransferases in established and primary GBM cells. (DOCX 925 kb)

## Abbreviations

ChIP: Chromatin immunoprecipitation
DnmTs: DNA methyltransferases
GBM: Glioblastoma multiforme
HOTAIRM1: HOXA transcript antisense RNA myeloid-specific 1
HOXA1: Homeobox A1
LncRNA: Long noncoding RNA
PRC2: Polycomb Repressive Complex 2
TCGA: The Cancer Genome Atlas
