# Supplementary material for: Over-expressed lncRNA HOTAIRM1 promotes tumor growth and invasion through up-regulating HOXA1 and sequestering G9a/EZH2/Dnmts away from the HOXA1 gene in glioblastoma multiforme
Source: J Exp Clin Cancer Res. 2018 Oct 30;37:265. doi: 10.1186/s13046-018-0941-x (PMC6208043; doi:10.1186/s13046-018-0941-x)
Supplement: Supplementary file 1 — Table S1. Primers for qRT-PCR (DOCX 20 kb) [file 13046_2018_941_MOESM1_ESM.docx]

Table S1 Primers for qRT-PCR

| Primer Name | Sequence (5' to 3') |
| --- | --- |
| GAPDH | S: GCACCGTCAAGGCTGAGAAC  A: TGGTGAAGACGCCAGTGGA |
| HOTAIRM1 | S: AGGGGGTTGAAATGTGGGTG  A: CTTGAAAGTGGAGAAATAAAGTGCC |
| HOXA1 | S: CCAGCATACATTTTCTGGTGGG  A: TACTTTCAAGGACAAGGGAGGG |
| HOXA2 | S: GCGCTGCTGGATTTGACTGA  A: CATTTCCCTTCGCTGTTTTGGT |
| HOXA3 | S: GCCCTTTCCTTCCTTCCTTTTC  A: CGCTATGATACAGCCATTCCAG |
| HOXA4 | S: CCCTCCCCATCTGGACCATAAT  A: TTGTTCCACCAGCCAGCATC |
| HOXA5 | S: GCGAGCCACAAATCAAGCA  A: ATTGTAGCCGTAGCCGTACCTG |
| HOXA6 | S: TCTGATAAGGACCTCAGTGGCG  A: TGCTGCTCGGGAGAAAAGTG |
| HOXA7 | S: TTGCACCCTAGAATCAATCCCT  A: CCTGCTAAGCAGAAGCTAACCC |
| HOXA9 | S: AACAAAGACCGAGCAAAAGACG  A: ATGTGGCCTGAGGTTTAGAGCC |
| HOXA10 | S: CAACTGGCTCACGGCAAAGA  A: TTCAGTTTCATCCTGCGGTTC |
| HOXA11 | S: CCCTCCCATTGAATCTCCTTTG  A: CCGTCTTTATTTTCCTTGTGCC |
| HOXA13 | S: TTACTAAGGACAAACGGAGGCG  A: GCAAAGCAACGAGTTCTGAAGC |
| HOTTIP | S: CCAATGTAAGTGTCGCCCAATA  A: GGCCAGTCAGGGAGAAGGTAAA |
