# Supplementary material for: Over-expressed lncRNA HOTAIRM1 promotes tumor growth and invasion through up-regulating HOXA1 and sequestering G9a/EZH2/Dnmts away from the HOXA1 gene in glioblastoma multiforme
Source: J Exp Clin Cancer Res. 2018 Oct 30;37:265. doi: 10.1186/s13046-018-0941-x (PMC6208043; doi:10.1186/s13046-018-0941-x)
Supplement: Supplementary file 2 — Figure S1. QPCR primer line amplification efficiency detection. (DOCX 394 kb) [file 13046_2018_941_MOESM2_ESM.docx]

A B
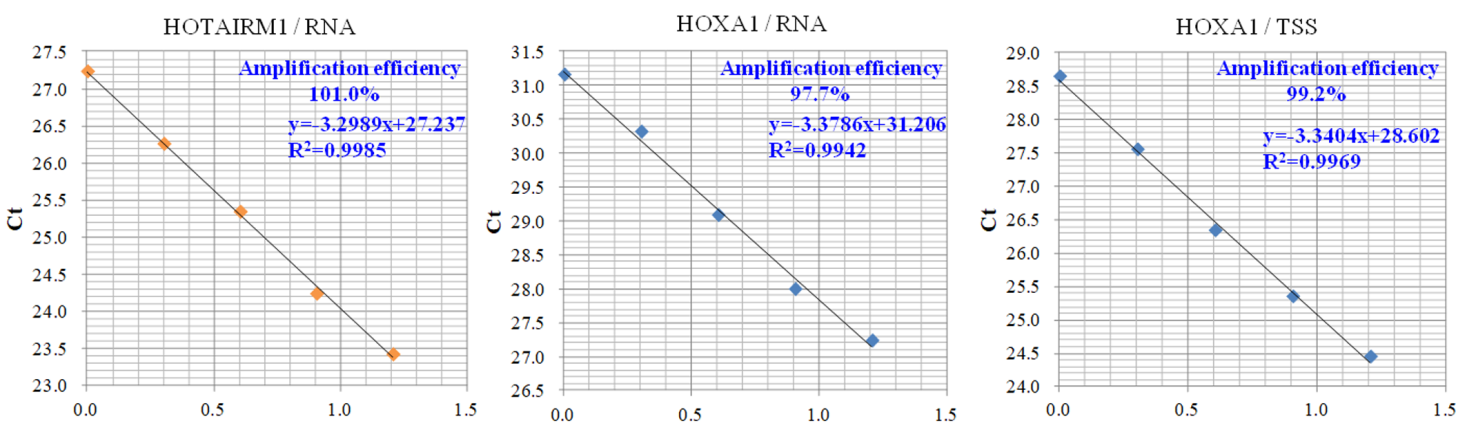
 C

**Figure S1**

QPCR primer line amplification efficiency detection.

The primer amplification efficiency of (A, B) HOTARIM1, HOXA1 RNA and (C) HOXA1 DNA was detected using cDNA or DNA template gradient dilution method respectively. The amplification efficiency were 97.7%~101.0%, approaching 100%.
