# Supplementary material for: Over-expressed lncRNA HOTAIRM1 promotes tumor growth and invasion through up-regulating HOXA1 and sequestering G9a/EZH2/Dnmts away from the HOXA1 gene in glioblastoma multiforme
Source: J Exp Clin Cancer Res. 2018 Oct 30;37:265. doi: 10.1186/s13046-018-0941-x (PMC6208043; doi:10.1186/s13046-018-0941-x)
Supplement: Supplementary file 3 — Table S2. Probes Sequence for HOTAIRM1 (DOCX 20 kb) [file 13046_2018_941_MOESM3_ESM.docx]

Table S2 Probes Sequence for HOTAIRM1

| Probe name | Probe Sequence (5' to 3') |
| --- | --- |
| HOTAIRM1_1 | tgcggaagccggcaaacttt |
| HOTAIRM1_2 | actaaaacggtgatccatca |
| HOTAIRM1_3 | gagcgccggggatttaaatg |
| HOTAIRM1_4 | caaatcggcctttgcagtcg |
| HOTAIRM1_5 | agaacgcagcttttgctctt |
| HOTAIRM1_6 | tttcattgaacggtggggag |
| HOTAIRM1_7 | ggactatggctggtttctgg |
| HOTAIRM1_8 | cttcctccgctaaatctcag |
| HOTAIRM1_9 | tgtttcttcataggtttgct |
| HOTAIRM1_10 | gaagttccaatgacaacgcg |
| HOTAIRM1_11 | gctcttaacagcaaaggctt |
| HOTAIRM1_12 | gcgggttgatttaagaacct |
| HOTAIRM1_13 | tgtaagcaacatgtgtgtgg |
| HOTAIRM1_14 | agaccgtgagaaaacgcagc |
| HOTAIRM1_15 | tgttgatgggttcaggcaaa |
| HOTAIRM1_16 | agtgtggttgattaatctcc |
| HOTAIRM1_17 | cataaatccctccacatttt |
| HOTAIRM1_18 | caaacacccacatttcaacc |
| HOTAIRM1_19 | ctccaggtcaataactaagt |
| HOTAIRM1_20 | gtttctttaataagctacca |
| HOTAIRM1_21 | tccaggaatgagtaacacgg |
| HOTAIRM1_22 | taaagtgcctacagaaaccc |
| HOTAIRM1_23 | ccaagctcttgaaagtggag |
| HOTAIRM1_24 | cagtctaagatttgggccaa |
| HOTAIRM1_25 | ggtaatagaggcagaattgg |
| HOTAIRM1_26 | aggttcaagccatagattta |
| HOTAIRM1_27 | aggatttgattttcagtgca |
| HOTAIRM1_28 | tcttctcctctttcttttta |
| HOTAIRM1_29 | ttttctttcttttttgcttc |
| HOTAIRM1_30 | gccaaaaaatgactagggct |
| HOTAIRM1_31 | gtcttcaatggacagcaaca |
| HOTAIRM1_32 | ttattaaggcggcatgttca |
