# Supplementary material for: Over-expressed lncRNA HOTAIRM1 promotes tumor growth and invasion through up-regulating HOXA1 and sequestering G9a/EZH2/Dnmts away from the HOXA1 gene in glioblastoma multiforme
Source: J Exp Clin Cancer Res. 2018 Oct 30;37:265. doi: 10.1186/s13046-018-0941-x (PMC6208043; doi:10.1186/s13046-018-0941-x)
Supplement: Supplementary file 4 — Table S3. siRNA oligonucleotides (DOCX 19 kb) [file 13046_2018_941_MOESM4_ESM.docx]

Table S3 siRNA oligonucleotides

| siRNA Name | Sequence (5' to 3') |
| --- | --- |
| siHOTAIRM1-1 | S: GGAGACUGGUAGCUUAUUATT  A: UAAUAAGCUACCAGUCUCCTT |
| siHOTAIRM1-2 | S: GCCGCCUUAAUAAAUGUAUTT  A: AUACAUUUAUUAAGGCGGCTT |
| siNegative Control  (siNC) | S: UUCUCCGAACGUGUCACGUTT  A: ACGUGACACGUUCGGAGAATT |
