# Supplementary material for: Over-expressed lncRNA HOTAIRM1 promotes tumor growth and invasion through up-regulating HOXA1 and sequestering G9a/EZH2/Dnmts away from the HOXA1 gene in glioblastoma multiforme
Source: J Exp Clin Cancer Res. 2018 Oct 30;37:265. doi: 10.1186/s13046-018-0941-x (PMC6208043; doi:10.1186/s13046-018-0941-x)
Supplement: Supplementary file 5 — Table S4. Primers for ChIP (DOCX 19 kb) [file 13046_2018_941_MOESM5_ESM.docx]

Table S4 Primers for ChIP

| Primer Name | Sequence (5' to 3') |
| --- | --- |
| HOXA1-1 | S: CGACTGCGCGTCACCTAGAC  A: GCCAATGGCTGAGCCTCCTGC |
| HOXA1-2 | S: AGCCCAGCCTCAGAACAGAGGA  A: GGAATCAGTGGGCCAGAGCTC |
| HOXA1-3 | S: GCCCCTCCAAGTCGAATTACAGA  A: AGGAGACGAGGGCAAGAAAAGAA |
| HOXA1-4 | S: TCCCCTCCAGAAAAAGAGGAAC  A: GTAGGAGGCGGGGGAGAGAGAT |
| HOXA1-5 | S: AACAACAATTTAACGACCTCAGCAG  A: GACACTTTACCCCAACGACACCT |
| HOXA2 | S: ATAGATCCTTGCAGATCAGGAGGC  A: CCAGCGAGGCTTTATTTACACTT |
| HOXA11 | S: CCCTGAGGTGGCTACAAAGAAAG  A: GGAGACGTAAGCTGACGTGGAAA |
