# Supplementary material for: Over-expressed lncRNA HOTAIRM1 promotes tumor growth and invasion through up-regulating HOXA1 and sequestering G9a/EZH2/Dnmts away from the HOXA1 gene in glioblastoma multiforme
Source: J Exp Clin Cancer Res. 2018 Oct 30;37:265. doi: 10.1186/s13046-018-0941-x (PMC6208043; doi:10.1186/s13046-018-0941-x)
Supplement: Supplementary file 6 — Table S5. Primers for methylation specific PCR (DOCX 18 kb) [file 13046_2018_941_MOESM6_ESM.docx]

Table S5 Primers for methylation specific PCR

| Primer Name | Sequence (5' to 3') |
| --- | --- |
| MSP-1 | S: AGGAAGAGAGTTTTAGAATAGAGGAGGTGGTTTGG  A:CAGTAATACGACTCACTATAGGGAGAAGGCTCAACTTTCTCACTTCCTCCATAAAA |
| MSP-2 | S: AGGAAGAGAGGGGAGTTTAGTTATTATGTTGGAGTTG  A:AGTAATACGACTCACTATAGGGAGAAGGCTAACTTCCCTTCTTCCAAAAAAATCT |
