# Supplementary material for: Over-expressed lncRNA HOTAIRM1 promotes tumor growth and invasion through up-regulating HOXA1 and sequestering G9a/EZH2/Dnmts away from the HOXA1 gene in glioblastoma multiforme
Source: J Exp Clin Cancer Res. 2018 Oct 30;37:265. doi: 10.1186/s13046-018-0941-x (PMC6208043; doi:10.1186/s13046-018-0941-x)
Supplement: Supplementary file 7 — Table S6. Primers for RNA-ChIP (DOCX 18 kb) [file 13046_2018_941_MOESM7_ESM.docx]

Table S6 Primers for RNA-ChIP

| Primer Name | Sequence (5' to 3') | Product length |
| --- | --- | --- |
| HOTAIRM1 | S: ACACTGAAAATGTGGAGGGAT  A: GACAGCAACAAAACAGAAAGC | 381 bp |
