# Supplementary material for: Over-expressed lncRNA HOTAIRM1 promotes tumor growth and invasion through up-regulating HOXA1 and sequestering G9a/EZH2/Dnmts away from the HOXA1 gene in glioblastoma multiforme
Source: J Exp Clin Cancer Res. 2018 Oct 30;37:265. doi: 10.1186/s13046-018-0941-x (PMC6208043; doi:10.1186/s13046-018-0941-x)
Supplement: Supplementary file 8 — Figure S2. Knockdown of HOTAIRM1 suppresses proliferation and induces apotosis of U87 cells. (DOCX 316 kb) [file 13046_2018_941_MOESM8_ESM.docx]

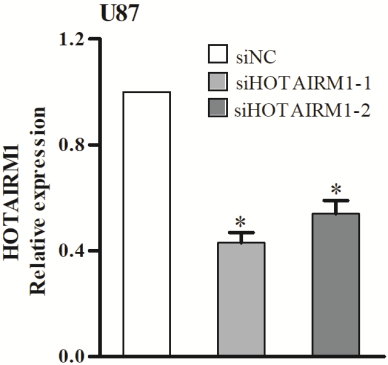

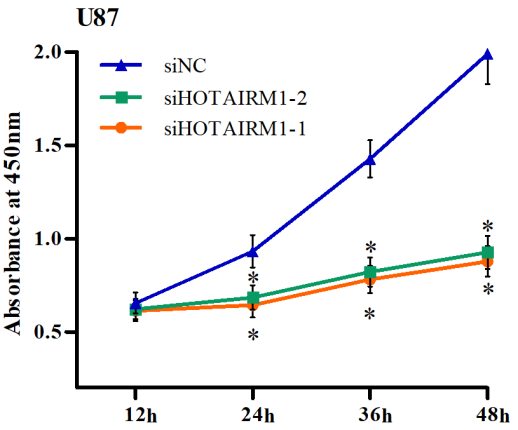
A  B


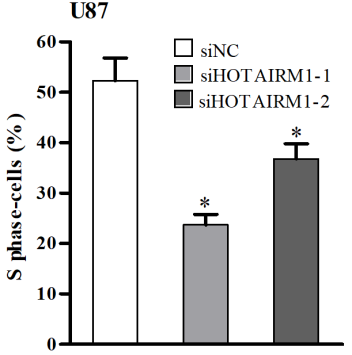

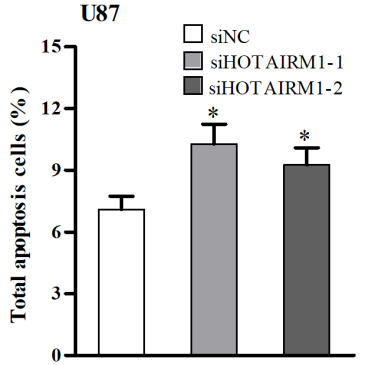
C D


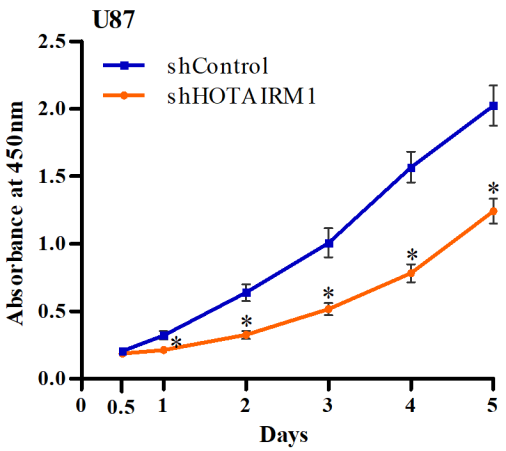


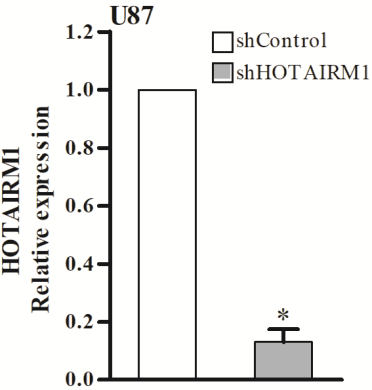
E F

**Figure S2**

Knockdown of HOTAIRM1 suppresses proliferation and induces apotosis of U87 cells. (A) The qRT-PCR analysis of HOTAIRM1 RNA levels at 24 h after siHOTAIRM1 treatment in U87 cell, with the *GAPDH* gene as an internal control. The siControl was a scrambled sequence with no homology to any known gene. B-D, After treatment with siHOTAIRM1 and siNC for 24 h, (B) U87 cell growth curve was determined by CCK-8 assay at various time points (12 to 48 h); (C) Flow cytometry cell cycle analysis showing changes in U87 cell proliferation; (D) Flow cytometry analysis showing cells apoptosis rate in U87 cell. Error bars represent the SEs of three independent experiments. (E) The qRT-PCR analysis of HOTAIRM1 RNA levels after transfection with lentivirus of shHOTAIRM1 or shControl with the *GAPDH* gene as an internal control in U87 cells. (F) After transfection with shHOTAIRM1 or shControl, U87 cell growth curve was determined by CCK-8 assay at different time point (0.5 to 5 day) , **P*<0.05.
