# Supplementary material for: Over-expressed lncRNA HOTAIRM1 promotes tumor growth and invasion through up-regulating HOXA1 and sequestering G9a/EZH2/Dnmts away from the HOXA1 gene in glioblastoma multiforme
Source: J Exp Clin Cancer Res. 2018 Oct 30;37:265. doi: 10.1186/s13046-018-0941-x (PMC6208043; doi:10.1186/s13046-018-0941-x)
Supplement: Supplementary file 9 — Figure S3. Knockdown of HOTAIRM1 suppresses proliferation and induces apotosis of established and primary GBM cells. (DOCX 202 kb) [file 13046_2018_941_MOESM9_ESM.docx]

A B


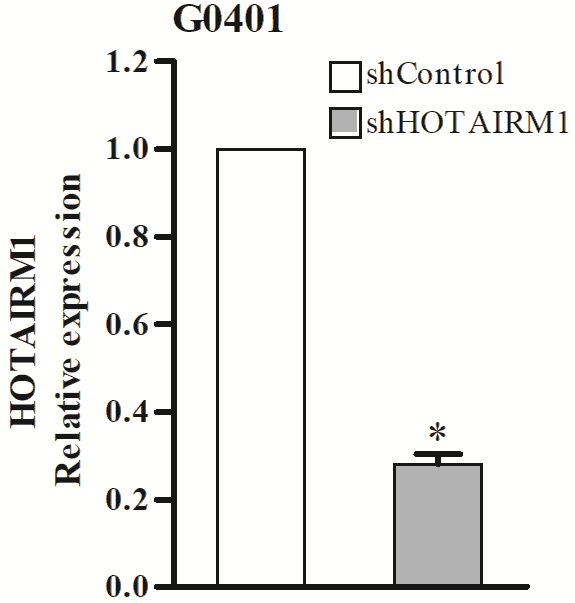

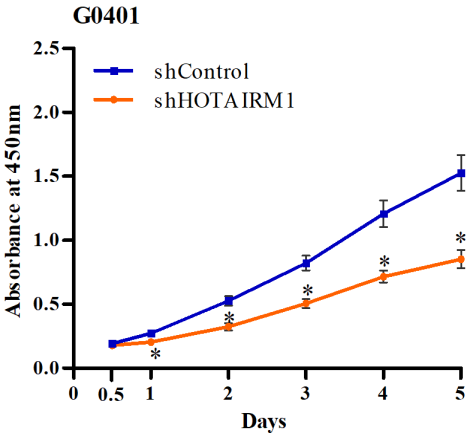


C D


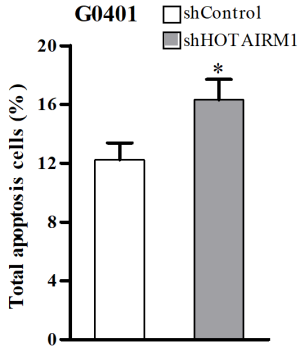

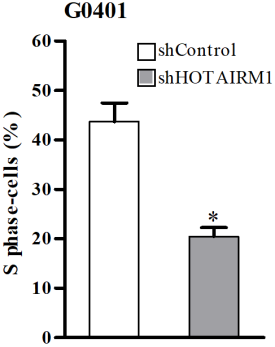


**Figure S3**

Knockdown of HOTAIRM1 suppresses proliferation and induces apotosis of established and primary GBM cells. (A) The qRT-PCR analysis of HOTAIRM1 RNA levels after transfection with lentivirus of shHOTAIRM1 or shControl with the *GAPDH* gene as an internal control in G0401 cells. (B) After transfection with shHOTAIRM1 or shControl, G0401 cell growth curve was determined by CCK-8 assay at different time point (0.5 to 5 day). After transfection with shHOTAIRM1 or shControl, (C) Flow cytometry cell cycle analysis showing changes in G0401 cells proliferation; (D) Flow cytometry analysis showing cells apoptosis rate in G0401 cells. Error bars represent the SEs of three independent experiments, **P*<0.05.
