# Supplementary material for: Over-expressed lncRNA HOTAIRM1 promotes tumor growth and invasion through up-regulating HOXA1 and sequestering G9a/EZH2/Dnmts away from the HOXA1 gene in glioblastoma multiforme
Source: J Exp Clin Cancer Res. 2018 Oct 30;37:265. doi: 10.1186/s13046-018-0941-x (PMC6208043; doi:10.1186/s13046-018-0941-x)
Supplement: Supplementary file 10 — Figure S4. Knockdown of HOTAIRM1 inhibits migration and invasion of established and primary GBM cells. (DOCX 203 kb) [file 13046_2018_941_MOESM10_ESM.docx]

A B


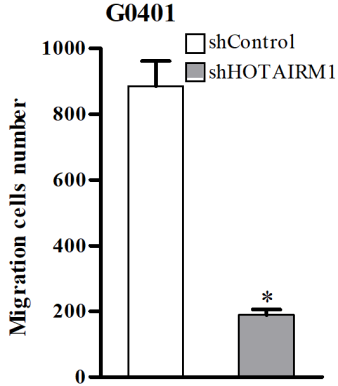

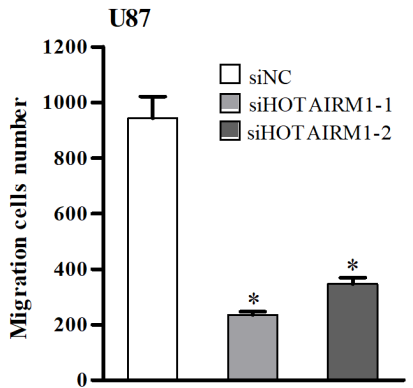


C D


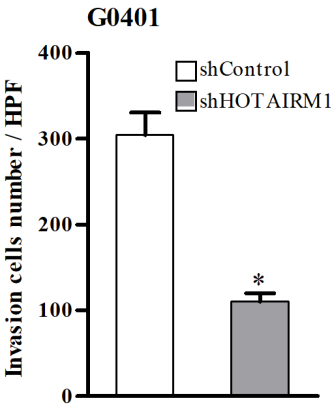

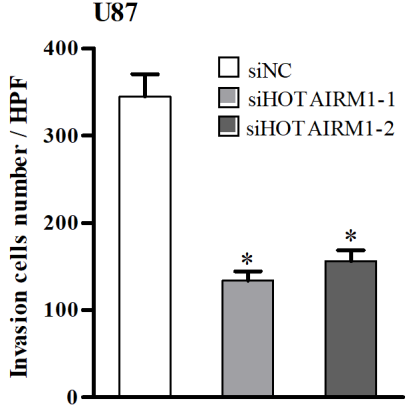


**Figure S4**

Knockdown of HOTAIRM1 inhibits migration and invasion of established and primary GBM cells. U87 cells were treated with siHOTAIRM1 or siNC for 24 h, and G0401 cells were transfected with shHOTAIRM1 or shControl, (A-B) cell migration was observed under microscope and migrated cell number was counted; (C-D) transwell cell invasion assay was performed, the invasive cells in members were stained with crystal violet and quantification under high-power field (HPF). Error bars represent the SEs of three independent experiments, **P*<0.05.
