# Supplementary material for: Over-expressed lncRNA HOTAIRM1 promotes tumor growth and invasion through up-regulating HOXA1 and sequestering G9a/EZH2/Dnmts away from the HOXA1 gene in glioblastoma multiforme
Source: J Exp Clin Cancer Res. 2018 Oct 30;37:265. doi: 10.1186/s13046-018-0941-x (PMC6208043; doi:10.1186/s13046-018-0941-x)
Supplement: Supplementary file 11 — Figure S5. HOTAIRM1 regulates HOXA1 RNA levels in established and primary GBM cells. (DOCX 297 kb) [file 13046_2018_941_MOESM11_ESM.docx]

A B


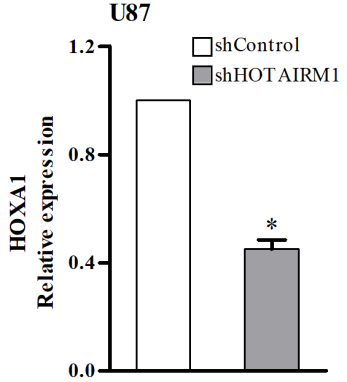

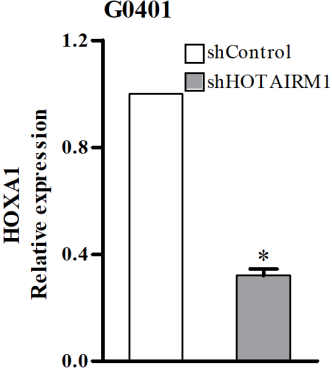


C


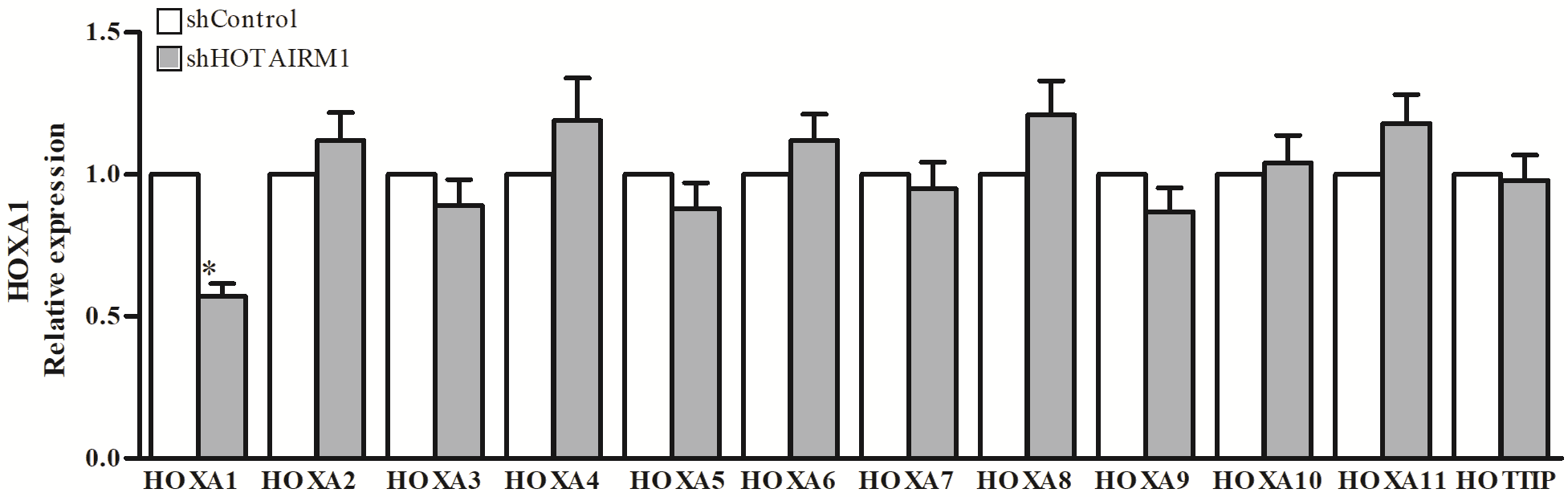


**Figure S5**

HOTAIRM1 regulates HOXA1 RNA levels in established and primary GBM cells. The RNA expression of HOXA1 was analyzed using qRT-PCR, (A) after transfection with shHOTAIRM1 or shControl in U87 cells and (B) in G0401 cells. (C) The RNAs expression of HOXA1, HOXA2, HOXA3, HOXA4, HOXA5, HOXA6, HOXA7, HOXA8, HOXA9, HOXA10, HOXA11 and HOTIP were analyzed using qRT-PCR, after transfection with shHOTAIRM1 or shControl. Error bars represent the SEs of three independent experiments, **P*<0.05.
