# Supplementary material for: Over-expressed lncRNA HOTAIRM1 promotes tumor growth and invasion through up-regulating HOXA1 and sequestering G9a/EZH2/Dnmts away from the HOXA1 gene in glioblastoma multiforme
Source: J Exp Clin Cancer Res. 2018 Oct 30;37:265. doi: 10.1186/s13046-018-0941-x (PMC6208043; doi:10.1186/s13046-018-0941-x)
Supplement: Supplementary file 12 — Figure S6. Knockdown of HOTAIRM1 increased H3K9me2 and H3K27me3 modifications in the promoter region of the HOXA1 gene in established and primary GBM cells. (DOCX 758 kb) [file 13046_2018_941_MOESM12_ESM.docx]

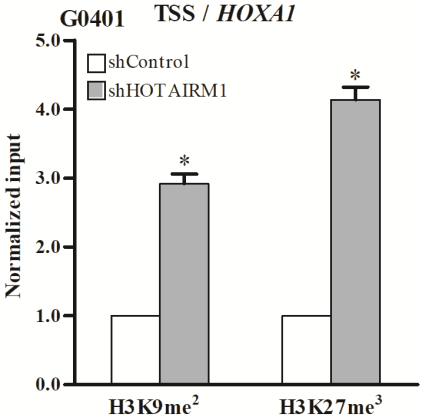

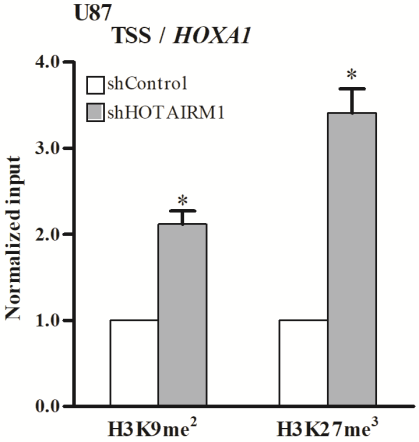
A B


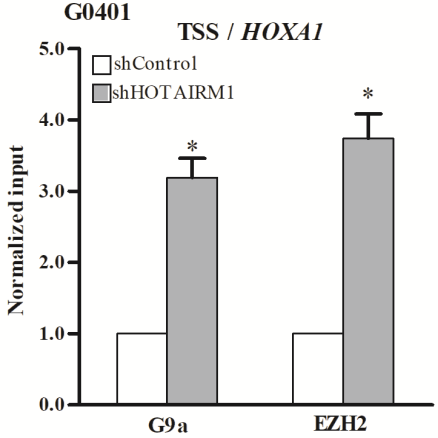

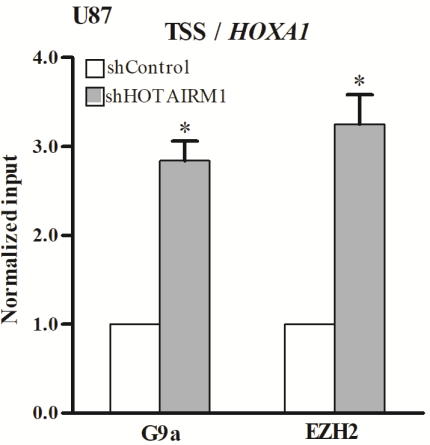
C D


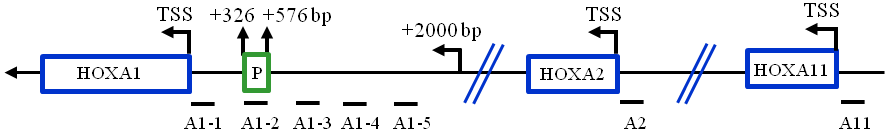


E


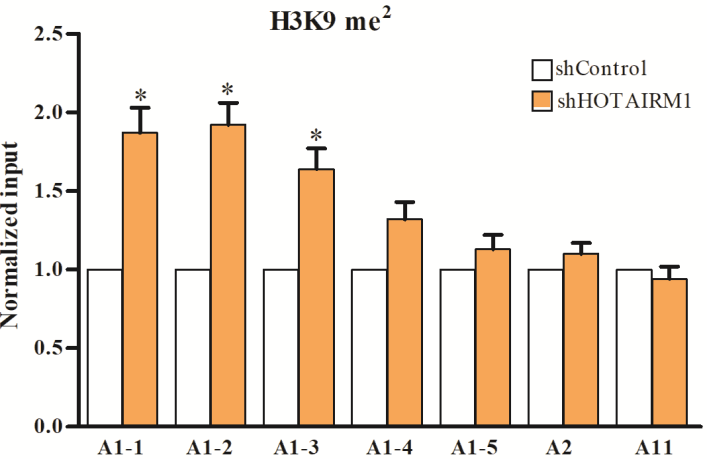
F G


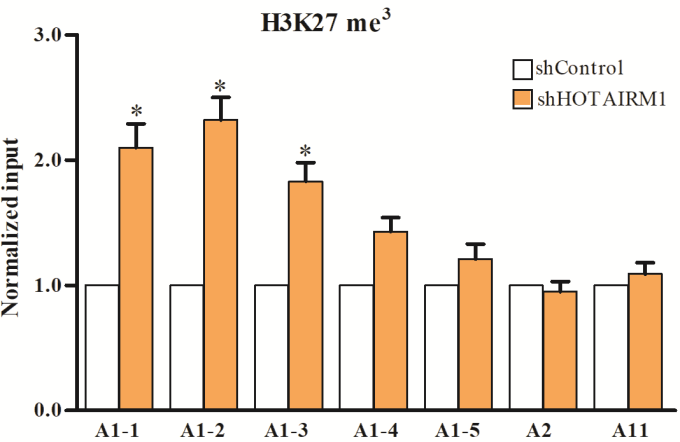


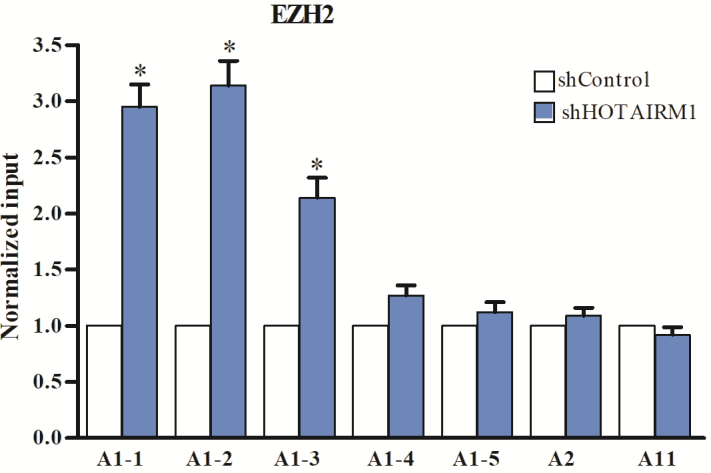
H I


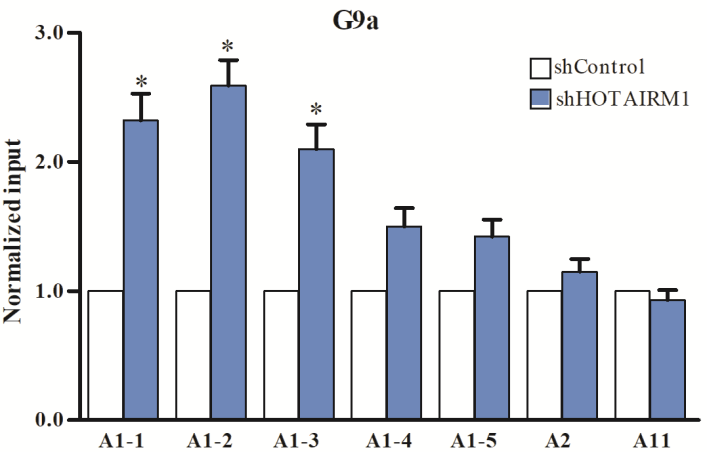


**Figure S6**

Knockdown of HOTAIRM1 increased H3K9me^2^ and H3K27me^3^ modifications in the promoter region of the *HOXA1* gene in established and primary GBM cells. Knockdown of HOTAIRM1, ChIP analysis of (A-B) H3K9me^2^ and H3K27me^3^ modifications of *HOXA1* gene promoter (A) in U87 cells and (B) in G0401 cells; (C-D) G9a and EZH2 enrichment in the *HOXA1* gene promoter (C) in U87 cells and (D) in G0401 cells. ChIP enrichment was measured using qPCR, normalized by the input DNA. (E) qPCR segments of ChIP in the TSS regions. ChIP analysis of (F) H3K9me^2^ and (G) H3K27me^3^ modifications of *HOXA1*, *HOXA2* and *HOXA11* TSS regions; (H) G9a and (I) EZH2 enrichment in the *HOXA1*, *HOXA2* and *HOXA11* TSS. Error bars represent the SE of three independent experiments, **P*<0.05.
