# Supplementary material for: Over-expressed lncRNA HOTAIRM1 promotes tumor growth and invasion through up-regulating HOXA1 and sequestering G9a/EZH2/Dnmts away from the HOXA1 gene in glioblastoma multiforme
Source: J Exp Clin Cancer Res. 2018 Oct 30;37:265. doi: 10.1186/s13046-018-0941-x (PMC6208043; doi:10.1186/s13046-018-0941-x)
Supplement: Supplementary file 13 — Figure S7. Knockdown of HOTAIRM1 induces CpG island methylation in the promoter region of the HOXA1 gene by increasing DNA demethyltransferases in established and primary GBM cells. (DOCX 925 kb) [file 13046_2018_941_MOESM13_ESM.docx]

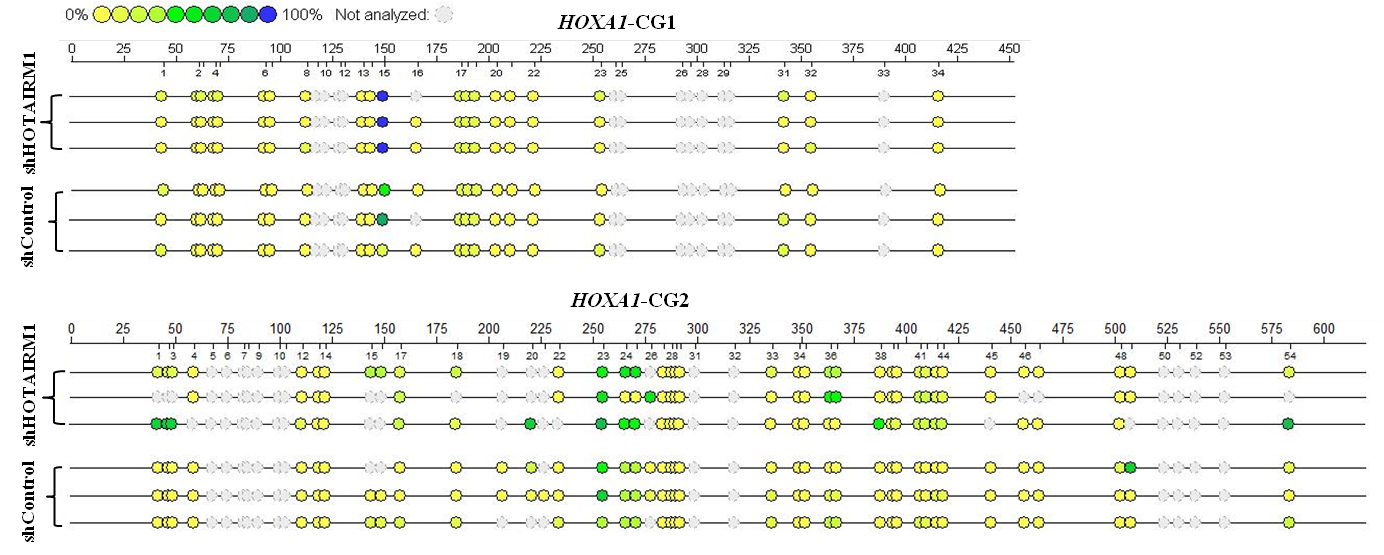
A


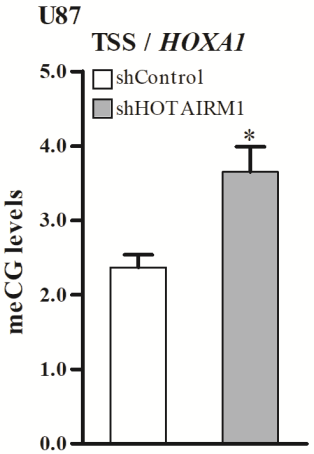

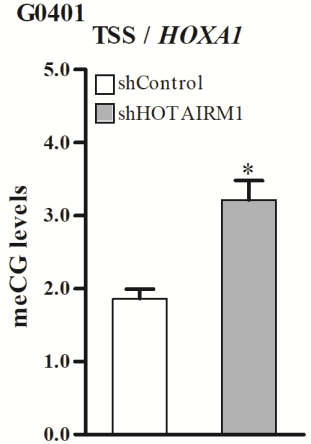
B C

D E


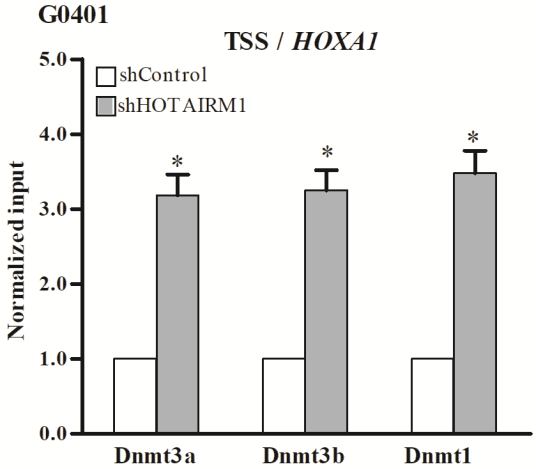

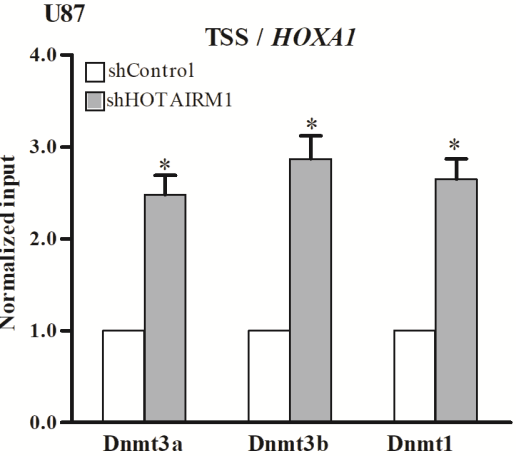


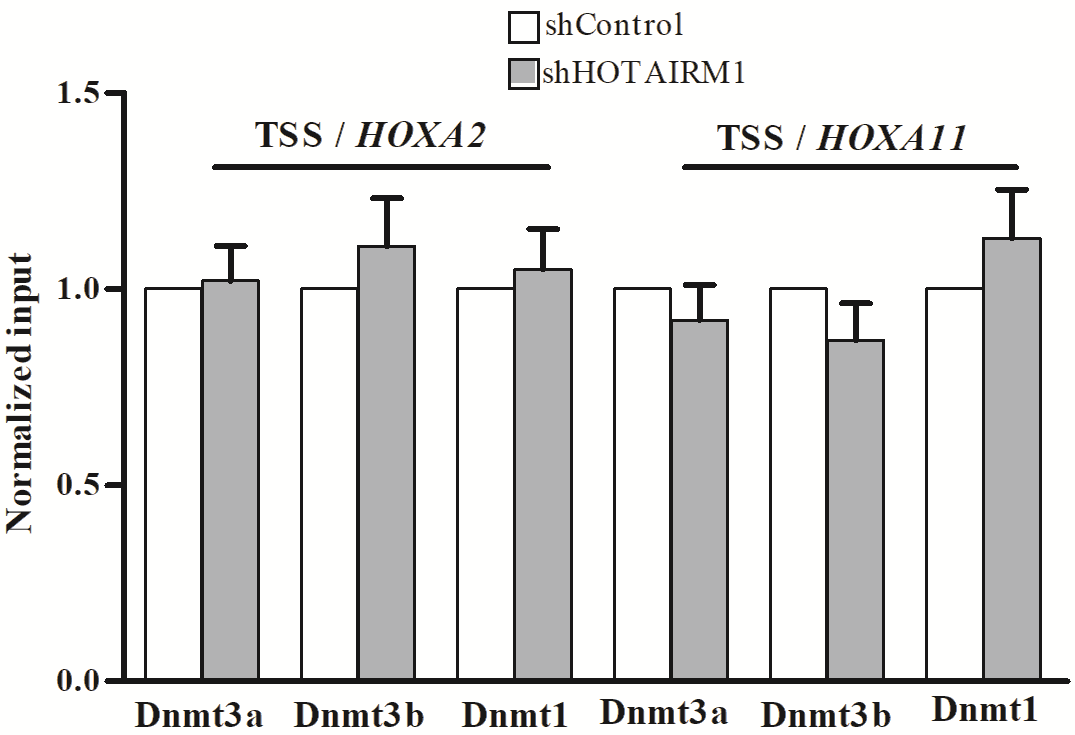
F

**Figure S7**

Knockdown of HOTAIRM1 induces CpG island methylation in the TSS region of the *HOXA1* gene by increasing DNA demethyltransferases in established and primary GBM cells. A, The schematic representation showing quantitative sequenom MassARRAY methylation analysis of CpG island methylation status in A172 cell after treatment with shHOTAIRM1 or shControl. B - C, The meCG levels in the *HOXA1* gene was measured to analyze DNA methylation status after knockdown of HOTAIRM1 (B) in U87 cells and (C) G0401 cells. D-E, After knockdown of HOTAIRM1, ChIP analysis of DNA methyltransferase binding in the HOXA1 TSS region (D) in U87 cells and (E) G0401 cells; (F) ChIP analysis of DNA methyltransferase binding in the HOXA2 and HOXA11 TSS region. Error bars represent the SE of three independent experiments, *P<0.05.
